# Cellulose modification for sustainable polymers: overcoming problems of solubility and processing†

**DOI:** 10.1039/d3su00317e

**Published:** 2024-01-04

**Authors:** Peter McNeice, Gert H. ten Brink, Ulrik Gran, Leif Karlson, Rolf Edvinsson, Ben L. Feringa

**Affiliations:** a Advanced Research Centre CBBC, Stratingh Institute for Chemistry, Faculty of Science and Engineering, University of Groningen Nijenborgh 4 Groningen 9747AG The Netherlands b.l.feringa@rug.nl; b Zernike Institute for Advanced Materials, University of Groningen Nijenborgh 4 Groningen 9747AG The Netherlands; c Performance Formulations, Nouryon SE-402 58 Göteborg Sweden

## Abstract

Two new water-soluble cellulose derivatives were prepared by a two-step transformation with 1,3-propane sultone, followed by either maleic or succinic anhydride, thereby converting cellulose into a more easily processable form. It was found that the solubility was dependent on both the degree of substitution and the chemical properties of the substituents. The water-soluble cellulose has a molecular weight greater than 100 000 g mol^−1^ and both the morphology and molecular weight can be tuned by varying the reaction conditions. Furthermore, the flexible, two-step nature of the process allows for expansion of this methodology in order to prepare cellulose analogues for different applications.

## Abstract 2

A flexible methodology to prepare water-soluble cellulose derivatives for easy processing is presented. The properties of the cellulose analogues are controllable, and the methodology enables further functionality to be added.

## Graphical abstract

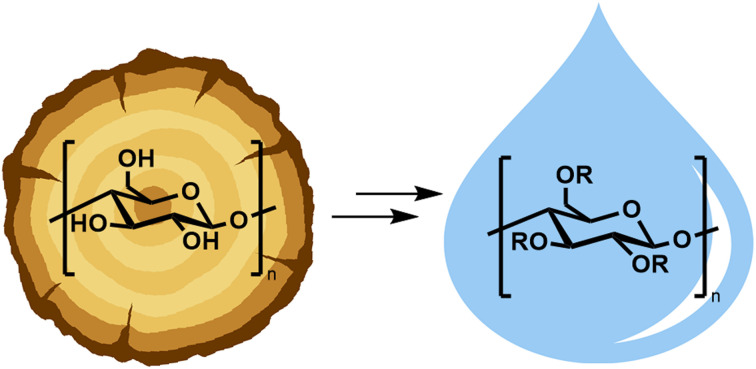

**Sustainability spotlight**

According to UN Sustainability Development Goal 12, there is an urgent need to reduce our reliance on fossil feedstocks in order to produce materials. The use of natural starting materials would help achieve this goal. Cellulose has excellent potential for this, as it is the most abundant renewable polymer. However, due to the challenge of solubility, converting cellulose into useful materials currently involves the use of toxic solvents and produces harmful waste. We overcome these problems by modifying cellulose to make it water-soluble. This benign method allows cellulose to be used as a polymer feedstock to contribute to the UN Sustainability Development Goal 12, whilst bringing the processing in line with the Principles of Green Chemistry.

## Introduction

Reducing the reliance on fossil feedstocks will help achieve a more sustainable society, as set out in the UN's Sustainable Development Goal 12.^1^ Currently, 90% of the feedstock for the synthesis of polymers, and in particular plastics, is reliant on oil and gas.^2^ It is estimated that 4–8% of the total oil produced is used in the manufacture of plastics. The production of plastics has increased 20-fold since 1964 and is expected to almost quadruple by 2050. Therefore, to meet the demand for plastics and polymers, whilst reducing the consumption of oil and gas, new methods to synthesise these materials must be developed. One potential solution is to use biomass as a feedstock for polymer production.

Cellulose is a clear choice as a future sustainable polymer feedstock. It is the most abundant biorenewable and biodegradable resource on earth, with an annual growth of 1.5 × 10^12^ tons.^3,4^ A major benefit of using cellulose as a feedstock rather than other biomass, is that it can be obtained from waste,^3–5^ and therefore does not compete with food production. Furthermore, highly pure nanocellulose can be produced by bacteria on an industrial scale,^6,7^ making cellulose an extremely promising green resource.

As shown by several recent reviews,^8–15^ the number and range of applications for cellulose based materials is expanding. These include food packaging,^8^ biomedical applications,^9–11^ water purification,^12^ photonic materials and pigments,^13,16,17^ and as an additive to alter the properties of polymer composites.^14,15^ However, a limiting factor to developing useful materials from cellulose is the difficulty in its processing.

**Fig. 1:**
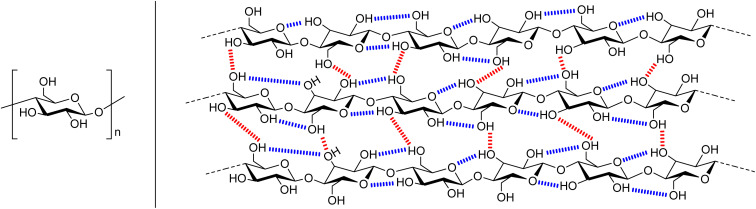
A representation of the structure of cellulose. Left is the anhydroglucose unit (AGU) and right shows the inter- (red) and intramolecular (blue) hydrogen bonds.

**Fig. 2:**
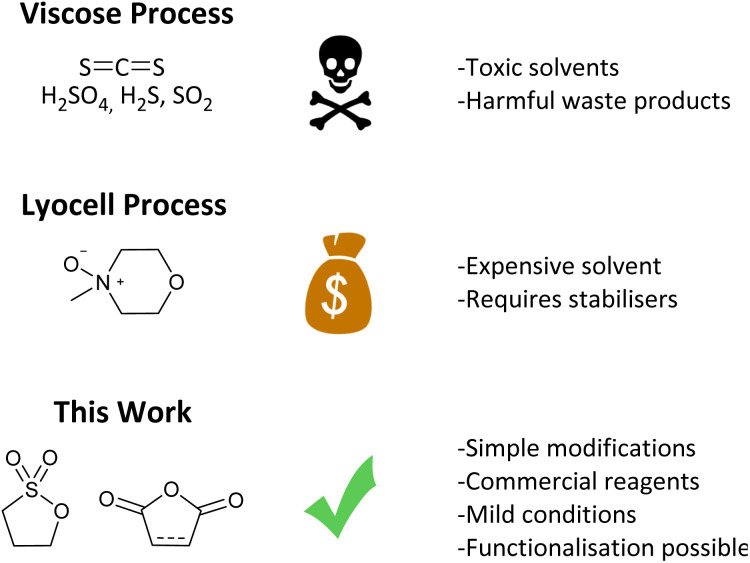
A comparison of industrial cellulose processing with this work.

Cellulose is a bio-polymer with up to 10 000 anhydroglucose (AGU) monomer units (Fig. 1, left),^4^ so it could be envisaged that the conversion of cellulose to other polymer materials would be facile. However, cellulose is insoluble in conventional solvents. Several factors account for this. Strong inter- and intra-molecular hydrogen bonding (Fig. 1, right), provides chain stiffness, and allows the linear chains to form sheet-like structures.^4^ Furthermore, hydrophobic interactions have been shown to have a significant contribution to cellulose–cellulose attraction, and therefore its insolubility.^18–20^ Dissolution and regeneration of cellulose is often necessary to produce fibres, films, and cellulose derivatives, as well as to assist in degradation for bio-refineries.^18^ However an alternate process is cellulose swelling. This is where a solvent causes changes in the molecular order of cellulose which can improve access to the fibres interior by increasing the surface area and porosity, and decreasing the crystallinity.^3,19,21^ One traditional industrial method for processing cellulose, the viscose process, relies on hazardous CS_2_, and produces waste in the form of H_2_S and SO_2_ (Fig. 2, top).^4^ The alternate, more environmental, Lyocell process involves regenerating cellulose from solutions of *N*-methylmorpholine-*N*-oxide by spinning.^4^ Drawbacks to this system are that the solvent is expensive and has poor thermal stability, with stabilisers required to prevent spontaneous exothermic reactions which would lead to cellulose degradation (Fig. 2, middle). Whilst ionic liquids have emerged as a milder way to dissolve and process cellulose,^22–28^ these have yet to find commercial use. Therefore, although using cellulose as a polymer feedstock can potentially improve sustainability, the use of toxic solvents, the production of waste, and the need for extra stabilisers means that current cellulose processing does not adhere to the Principles of Green Chemistry.^29^

Despite the environmental improvements offered by the Lyocell process, it is beneficial to have a form of cellulose which is itself soluble in conventional solvents, thereby enabling facile processing of cellulose for polymer synthesis. Derivatisation can be used to produce cellulose analogues,^3,4^ the most common of which, cellulose esters and ethers, are available commercially.^30–32^ Ethers are generally water soluble, and find use in the food and beverage, paint and coatings, textile, and construction industries as rheology modifiers,^3,30–33^ the mining industry to improve product yield,^3,30^ the oil and gas industry as drilling fluid,^3,30,31^ as well as being applied in tissue engineering^3,33^ and bio-sensing.^33^ Some esters, such as cellulose triacetate, can be water-soluble (depending on the degree of polymerisation) but many are not. Cellulose esters are used mainly to produce fibres, plastics, films, and coatings.^3,34,35^ Unfortunately, derivatisation processes can suffer from the same problems as described above for the viscose and Lyocell processes, namely the use of toxic or expensive solvents. New derivatisation methods to produce water-soluble cellulose in a more environmentally friendly manner are highly desirable to meet the UN's Sustainable Development Goals, whilst also following the Principles of Green Chemistry.

We present a simple, two-step modification of microcrystalline cellulose using organic compounds that render it water-soluble, whilst maintaining the backbone and polymer structure (Fig. 2, bottom). Analysis of these materials shows that both the degree of substitution and the nature of the substituent play a role in solubility, with a sulfonate group being essential. Furthermore, altering the second step allows the morphology (porous or plate-like) and molecular weight (35 000–243 000 g mol^−1^) of the material to be varied, whilst the inclusion of carboxylic acid and alkene moieties open up the potential for further modifications, making this a truly versatile process.

## Results and discussion

**Scheme 1:**
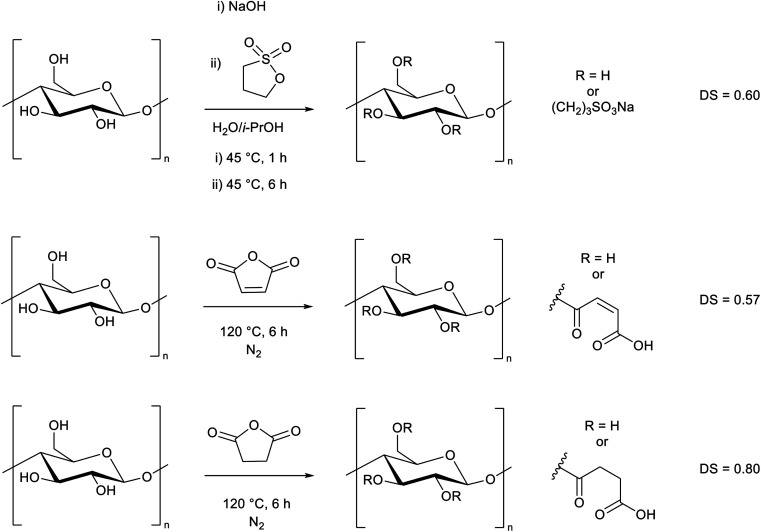
Modification of cellulose with 1,3-propane sultone, maleic anhydride, and succinic anhydride. The degree of substitution (DS) is shown (maximum DS = 3).

As a major reason for the lack of solubility of cellulose is strong hydrogen bonding from the alcohol groups, we selected these sites for modification, aiming to improve the solubility by disrupting the hydrogen bonding interactions. We decided to specifically target water solubility, and therefore selected cyclic molecules which would be ring-opened *via* a reaction with cellulose hydroxyl groups to provide moieties with high water affinity. To this end we chose 1,3-propane sultone, maleic anhydride, and succinic anhydride. It is well-known that maleic anhydride^36–40^ and succinic anhydride^41,42^ can be derived from nature. 1,3-Propane sultone can also be derived from glycerol *via* allyl alcohol reacted with sodium sulfite.^43–45^ The toxicity of 1,3-propane sultone should not to be an issue as it is used as a reagent rather than solvent, and the final product is non-toxic.^46^ We adapted literature procedures to react these species with microcrystalline cellulose (Scheme 1, ESI Sections S2.2 and S2.3†).^47,48^ While the presence of water may lead to side reactions in the 1,3-propane sultone step, it has been reported to be essential for the reaction to proceed.^47^ A similar degree of substitution (DS) was obtained in each reaction, as determined by elemental analysis (ESI section S3†). The slightly higher DS from succinic anhydride (DS = 0.80) could be due to a preferential adsorption at the cellulose interface compared to maleic anhydride (DS = 0.57), as reported by Sehaqui *et al.*^49^ The maximum DS is 3, which represents all 3 OH groups in a glucose unit being substituted. We expect the primary alcohol to be substituted preferentially, but other substitution patterns have been reported using related methods.^3^ Despite a decrease in crystallinity (ESI Fig. S5†), in all three cases (Scheme 1) the cellulose derivatives remained insoluble in water.

**Fig. 3:**
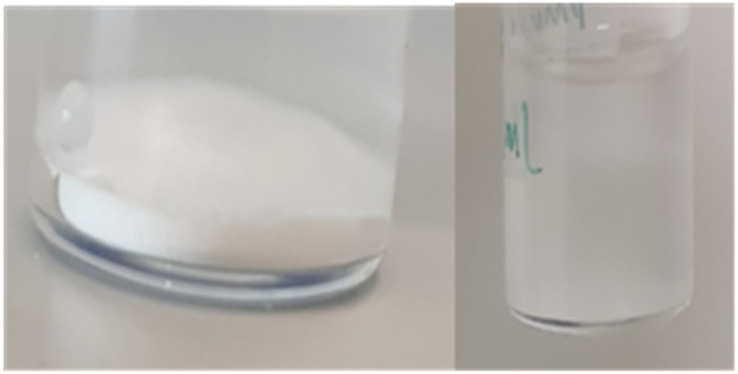
The interaction of sulfonated cellulose with water. Left: water-repellent behaviour, and right: the formed suspension.

Although the cellulose was insoluble, we noticed a different qualitative behavior after modification with 1,3-propane sultone. Whilst the unmodified cellulose could be easily dispersed in water, the sulfonated cellulose seemed at first to repel water, before slowly absorbing it to form a suspension (Fig. 3).

**Scheme 2:**
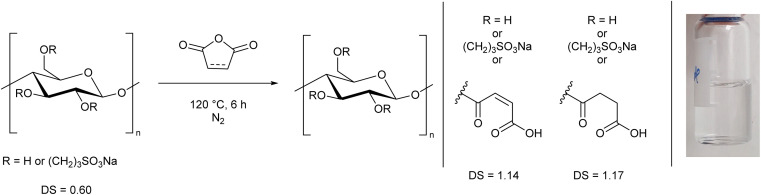
Synthesis of water-soluble cellulose derivatives from the reaction of sulfonated cellulose (Scheme 1) with either maleic or succinic anhydride. The DS and an example solution are shown.

Based on this observation, we used the sulfonated cellulose as a substrate for further reactions. Reacting this species with maleic or succinic anhydride, using the anhydride as both solvent and reagent, led to a DS greater than 1.1, and notably it allowed the cellulose to fully dissolve in water (Scheme 2, ESI Section S2.4†), thereby demonstrating a simple two-step process to prepare a water-soluble form of cellulose. We performed the reaction in this order to prevent removal of the anhydride group *via* cleavage of the ester linkage during the sultone reaction. ^1^H NMR spectra of these cellulose analogues clearly show the AGU unit and the sultone CH_2_ groups (ESI Section S5†). The maleic and succinic resonances are less distinct, presumably due to substitution at different alcohol groups along the chain.

**Scheme 3:**
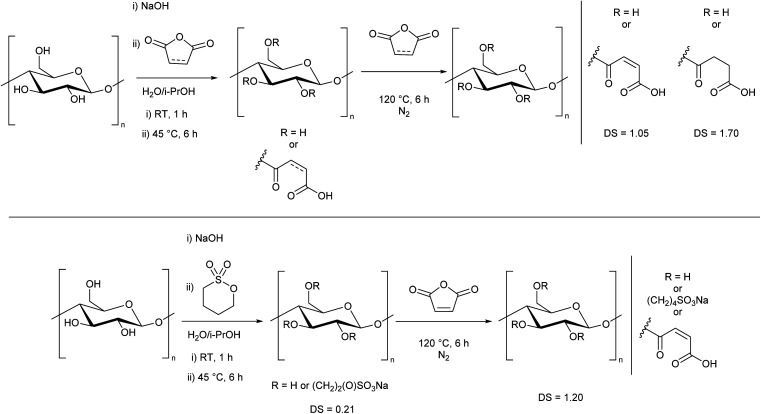
Alternate syntheses of modified cellulose where 1,3-propane sultone is replaced with (top) maleic or succinic anhydride, or (bottom) 1,4-butane sultone. These modified celluloses were not water-soluble.

We then determined that the water solubility was related to both the DS and the nature of the substituent. Zero, or low sulfonate incorporation did not yield water-soluble cellulose, despite a high DS. By performing analogous syntheses where 1,3-propane sultone was replaced by either maleic or succinic anhydride, degree of substitutions of 1.05 and 1.70 were achieved, respectively (Scheme 3, top, ESI Section 2.5†). However, despite having similar DS as the water-soluble derivatives, these analogues were not water-soluble, showing the importance of the ionic sulfonate group. This also demonstrates that the treatment with NaOH in the first step is not a defining factor in the dissolution, but rather the means to facilitate the efficient reaction of cellulose with 1,3-propane sultone.

Furthermore, when 1,4-butane sultone was used in place of 1,3-propane sultone (Scheme 3, bottom, ESI Section S2.2†), despite a higher total DS of 1.20, the cellulose remained insoluble, presumably due to a low sultone incorporation (DS 0.21) which shows that a significant amount of sulfonate group is essential to produce water-soluble cellulose with this method.

Analysis of the cellulose analogues by XRD show that all species have reduced crystallinity compared to microcrystalline cellulose (Fig. 5), however, cellulose without a sulfonate group (Fig. 5, red), or with only a small amount of sulfonate (Fig. 5, grey), has a higher crystallinity than water-soluble cellulose (Fig. 5, blue). Higher crystallinity is associated with stronger hydrogen bonding, dispersion forces, and electrostatic attractions, which reduce access of reactants to the cellulose surface,^50–52^ thereby preventing cellulose solubility by hindering interaction with water molecules.

**Fig. 4:**
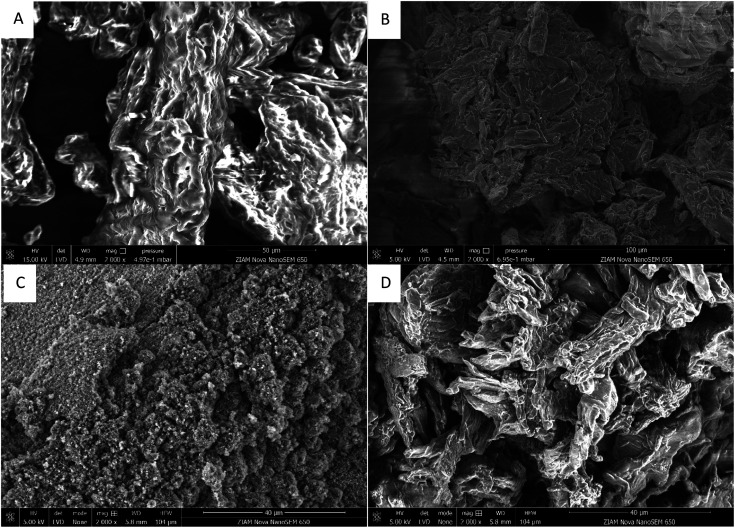
SEM images at 2000× magnification of microcrystalline cellulose (A), 1,3-propane sultone modified cellulose (B), water-soluble cellulose with maleic anhydride substituents (C), and water-soluble cellulose with succinic anhydride substituents (D).

It has previously been reported that cellulose bearing sulfonate groups can be water-soluble,^47,53–55^ however, the solubility was strongly dependant on the reaction conditions (temperature, time, concentration, solvent, *etc.*). These derivatives have low importance commercially, with carboxymethylcellulose being much more widely used.^3^ A major benefit of our two-step process is that different properties can be conferred onto the final cellulose material depending on the second step. This is clearly seen in the SEM images showing the morphology of the modified cellulose (Fig. 4). A more porous material is formed when maleic anhydride is used in the second modification step compared to succinic anhydride, where the natural cellulose morphology is largely maintained (Fig. 4C and D). We believe this is due to the lower first p*K*_a_ of maleic acid (1.8)^56^ compared to succinic acid (4.2).^56^ Further modulation of the polymer properties is possible by varying the reaction conditions (time and excess of anhydride). This is seen in the molecular weight obtained. For maleic anhydride, increasing the reaction time, or increasing the cellulose concentration in the maleic anhydride, reduces the molecular weight of the polymer (Table 1, entries 1–3). However, the opposite trend is observed with succinic anhydride (Table 1, entries 4–6). This could be due to a lower amount of cellulose decomposition when succinic anhydride is used as modifier, due to higher p*K*_a_ of the corresponding acid, or some condensation reactions may occur with a longer reaction time or more concentrated reaction mixture,^48^ cross-linking the succinate groups and increasing the molecular weight. Using this method, we reach the remarkable value of 242 500 g mol^−1^. The ability to control the molecular weight allows for the design of materials for different applications. Despite the different DS and molecular weights, there was little difference in thermal stability between the different modified celluloses (ESI Section S9†). All cellulose analogues containing a sulfonate group have a lower decomposition temperature than microcrystalline cellulose, which can be expected due to their lower crystallinity.^57^ However, they remained stable up to almost 200 °C, which is suitable for many applications, including traditional cellulose polymer processing.^58^ It should be emphasised that high temperature processing will not be necessary due to the water solubility of the cellulose. Furthermore, sulfonate modified cellulose did not decompose to 100% by mass, unlike unmodified cellulose, opening the possibility of these modified celluloses functioning as flame retardants.^59^

**Fig. 5:**
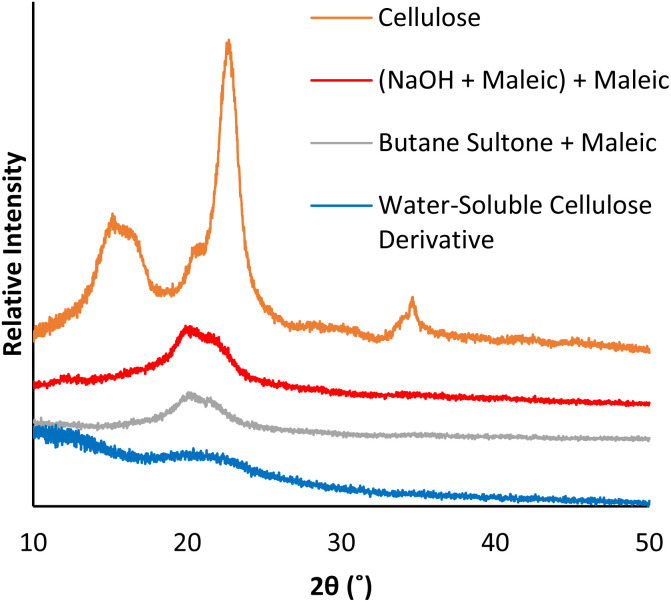
XRD patterns of cellulose, and differently modified cellulose analogues (from Schemes 2 and 3).

**Table 1:** The effect of reaction conditions on the DS and molecular weight of modified cellulose

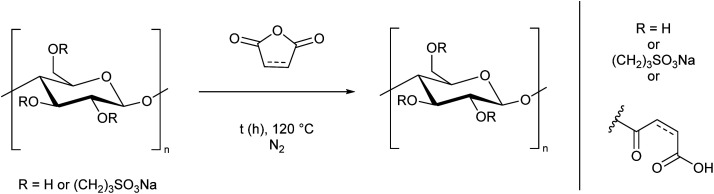				
Entry	Reactant	Reaction conditions	DS^a^	*M*_w_ b (g mol^−1^)
1	Maleic anhydride	Sulfonated cellulose (**100 mg, 5 wt%**), anhydride (2 g), 120 °C, **6 h**	1.14	145 900
2	Maleic anhydride	Sulfonated cellulose (**100 mg, 5 wt%**), anhydride (2 g), 120 °C, **16 h**	1.05	93 830
3	Maleic anhydride	Sulfonated cellulose (**500 mg**), anhydride (**1 g, 3 equiv.**^c^), 120 °C, **6 h**	1.08	34 550
4	Succinic anhydride	Sulfonated cellulose (**100 mg, 5 wt%**), anhydride (2 g), 120 °C, **6 h**	1.17	109 400
5	Succinic anhydride	Sulfonated cellulose (**100 mg, 5 wt%**), anhydride (2 g), 120 °C, **16 h**	1.02	130 700
6	Succinic anhydride	Sulfonated cellulose (**500 mg**), anhydride (**1 g, 3 equiv.**^c^), 120 °C, **6 h**	1.08	242 500

a Determined *via* elemental analysis and back titration.

b Determined *via* gel permeation chromatography using a Pullulan calibration.

c Based on the molecular weight of the anhydroglucose unit (162.14 g mol^−1^) of cellulose.

## Outlook

Using cellulose as a feedstock for polymers is in alignment with the UN's Sustainable Development Goals.^1^ However, as discussed in the introduction, processing cellulose is usually not environmentally benign. In this work we demonstrate a simple process which facilitates greener processing of cellulose. The water-soluble cellulose derivatives prepared here could be used in one of the many applications set out in the introduction. Aside from these applications, the water solubility will aid processing to develop new materials. For example, after cross-linking, the presence of sulfonate groups opens the possibility of preparing proton exchange membranes.^60–64^ Furthermore, the carboxylic acid and alkene moieties provided by the anhydrides offer scope for further reactivity, and studies along this line are in progress. This could lead to more functional groups being added to the cellulose in order to tune the physical properties. For example, cross-linking the maleic anhydride double bond could convert the cellulose from a polymer into a coating, as demonstrated for short chain oligomers of glucose with maleate substituents.^65^ Composite materials prepared using water-soluble cellulose could be biodegradable, which would contribute to their sustainability.^3–5,8,66^

**Scheme 4:**
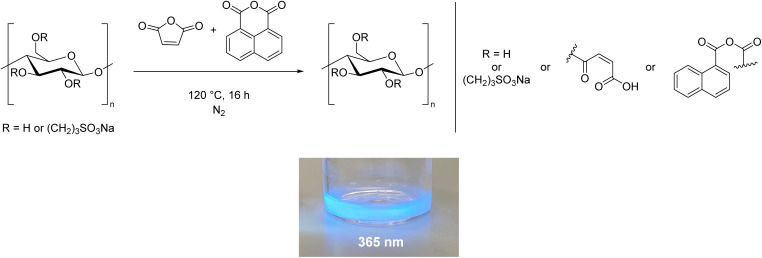
The preparation of a fluorescent cellulose derivative using our methodology.

We believe an additional advantage to the modification method reported here is the flexibility offered by the unique two-step process, which allows the properties of cellulose to be tuned based on the substituent added in the second step. Cellulose is intrinsically chiral but it is difficult to transfer this into the bulk phase.^67–69^ The addition of chiral groups could aid this process. Replacing maleic and succinic anhydride with tartaric acid derivatives, or performing a ring-opening polymerisation with lactide^70,71^ would add chiral side chains to the cellulose. This could exploit the inherent chiral properties in solution, for instance, to create liquid crystals.^67–69,72^ Another potential use is to prepare fluorescent cellulose materials using our procedure. Including compounds such as 1,8-naphthalic anhydride, or perylene tetracarboxylic acid in the synthesis could impart fluorescent properties to the cellulose, as reported by Tian *et al.*,^73^ opening the possibility of applications in sensing, printing and anti-counterfeiting. Our preliminary results in this area demonstrate that it is possible to prepare fluorescent cellulose derivatives using the methodology developed here (Scheme 4, ESI Section 2.6†).

We therefore present not only two specific water-soluble cellulose derivatives, but also a methodology which we anticipate will allow for the preparation of further water-soluble cellulose analogues with tailored functionality.

## Conclusions

We report a two-step modification of cellulose to produce a water-soluble polymer. Cellulose is first reacted with 1,3-propane sultone, and then with either maleic or succinic anhydride, making it water-soluble. Both the degree of substitution (DS) and the nature of the substituent were found to influence the water solubility, with a sulfonate group being essential in our process. The morphology of the final material depended on the treatment, with maleic anhydride producing a more porous structure, and succinic anhydride a plate-like structure. Remarkably, the molecular weight of the cellulose under our standard conditions was greater than 100 000 g mol^−1^ and could be increased or decreased depending on the reaction conditions (Table 1). Unlike current industrial methods to dissolve cellulose, we do not require toxic or unstable solvents. As well as the common uses for water-soluble cellulose, the methodology introduced here could be expanded, offering attractive prospects for different functionalities to be added in order to alter the properties, and the end use, of the cellulose.

## Conflicts of interest

There are no conflicts to declare.

## Supplementary Material

SU-002-D3SU00317E-s001
