# Supplementary material for: Cellulose modification for sustainable polymers: overcoming problems of solubility and processing
Source: RSC Sustain. 2024 Jan 4;2(2):369–76. doi: 10.1039/d3su00317e (PMC10849079; doi:10.1039/d3su00317e)
Supplement: SU-002-D3SU00317E-s001 [file SU-002-D3SU00317E-s001.pdf]

Electronic Supplementary Material (ESI) for RSC Sustainability.
This journal is © The Royal Society of Chemistry 2023

Cellulose Modification for Sustainable Polymers: Overcoming problems of solubility and processing

Peter McNeice,[a] Gert H. ten Brink,[b] Ulrik Gran,[c] Leif Karlson,[c] Rolf Edvinsson,[c] Ben L. Feringa[a,*]

a. Advanced Research Centre CBBC, Stratingh Institute for Chemistry and Zernike Institute for Advanced Materials, Faculty of Science and Engineering, University of Groningen, Nijenborgh 4, Groningen, 9747AG, Netherlands.
b. Zernike Institute for Advanced Materials, University of Groningen, Nijenborgh 4, Groningen 9747AG, Netherlands
c. Performance Formulations, Nouryon, SE-402 58 Göteborg, Sweden.

34 Pages: S1-S34

24 Figures: S1-S24

30 Spectra: S1-S30

Contents

S1 General information S2
S2 Transformations of cellulose S2
S2.1 NaOH treatment of cellulose S2
S2.2 Reaction of cellulose with 1,3-propane sultone S3
S2.3 Reaction of cellulose with cyclic anhydrides S3
S2.4 Preparation of water-soluble cellulose S3
S2.5 NaOH test reactions S5
S2.6 Preparation of a fluorescent cellulose derivative S6
S3 Degree of Substitution (DS) Calculation S6
S3.1 Homo-Substituted Cellulose S6
S3.2 Hetero-substituted cellulose S8
S4 Infrared (IR) Spectra S9
S5 Nuclear Magnetic Resonance (NMR) spectra S17
S6 Gel Permeation Chromatography (GPC) Data S24
S7 X-Ray Diffraction (XRD) Analysis S25
S8 Field Emission Scanning Electron Microscope (FE-SEM) S26
S8.1 Cellulose S26
S8.2 Sulfonated Cellulose S28
S8.3 Water-soluble cellulose from 6 h maleic anhydride reaction S30
S8.4 Water-soluble cellulose from 6 h succinic anhydride reaction S32
S9 Thermogravimetric Analysis (TGA) S34
S10 References S34

S1 General information

Cellulose (Microcrystalline, powder, product code 435236), succinic anhydride (≥99%), and sodium hydroxide pellets (≥97%) were purchased from Sigma Aldrich. 1,3-propane sultone (>99.0%) and maleic anhydride (>99.0%) were purchased from TCI Europe. All solvents were obtained from commercial suppliers and used as received.

Infrared (IR) spectra were recorded on a FT-IR Spectrum Two with a KBr window and a $LiTaO_3$ detector from PerkinElmer. Assignment was based on literature values.[1] Proton **nuclear magnetic resonance** (^1H NMR) spectra and carbon nuclear magnetic resonance (^{13}C NMR) spectra were recorded at 400 MHz using 400/54 Premium Shield from Agilent Technologies. The chemical shifts were reported in ppm and referenced to the solvent signal of partly deuterated D_2O at 4.79 ppm.[2] Signal multiplicities are reported as singlet (s), doublet (d), triplet (t), quartet (q), quintet (quin), sextet (s) and multiplet (m). **Gel permeation chromatography** (GPC) was performed on a Vanquish from Thermo Scientific equipped with a 2 PSS Suprema column: (100; 1000 Å, 300 x 8 mm 10 µm) using aqueous 0.05 M NaNO3 as eluent. The column was operated at 40 °C with an injection volume of 10 µL and a flow rate of 1 mL/min. Detection was accomplished using a RID detector. The molecular weight was calculated with ethylene glycol as an internal standard using a Pullulan calibration. **X-ray diffraction** (XRD) patterns were recorded on a D8 Advance from Bruker using Cu K α radiation (0.154 nm), measuring between 2θ of 10 ° and 50 ° at a step rate of 0.020 °/s. **Field Emission Scanning Electron Microscope** (FE-SEM) images were recorded on a Nova NanoSEM, without sputtering, using a Low Vacuum Detector. **Thermogravimetric analysis** (TGA) was performed on a TGA5500 from TA instruments, using a platinum pan. Measurements were carried out using N_2 as the carrier gas, the temperature range was between 25 and 700 °C with a ramp rate of 10 °C/min.

S2 Transformations of cellulose

For water-soluble cellulose, NMR, IR, XRD and GPC analyses were performed. For cellulose analogues that were insoluble in water, characterisation is limited to XRD and IR.

S2.1 NaOH treatment of cellulose

Adapted from Natus and Goethals[3]

In a 100 mL round bottomed flask, cellulose (2.0 g, 12 mmol, RMM assumed to be 162.14 gmol^{-1} based on the anhydroglucose unit, AGU) was suspended in *i*-PrOH (15 mL) and water (1.8 mL). Then 30 w/w NaOH (2.41 mL, 6.1 mmol, wt.% NaOH of total solution = 4.5) was added and stirred at 45 ˚C for 7 h and then overnight at RT. The suspension was poured into methanol (60 mL). The suspension was filtered with suction and washed with MeOH before drying in a desiccator under vacuum at RT overnight. A white solid, 2.142 g was recovered.

IR v_{max}/cm^{-1}: 3365br (O-H), 2879 (C-H), 1643 (O-H), 1368 (C-H), 1155 (C-O-C), 1034 (C-O-C), (Spectrum S1).

To test for water-soluble fractions, the filtrate was dried *via* rotary evaporation to leave a white solid. This material was analysed by ^1H NMR and IR. No cellulose was present.

S2.2 Reaction of cellulose with 1,3-propane sultone

Adapted from Natus and Goethals.[3]

In a 100 mL round bottomed flask, cellulose (2.0 g, 12 mmol, RMM assumed to be 162.14 $gmol^{-1}$ based on AGU) was suspended in *i*-PrOH (15 mL) and water (1.8 mL). Then 30 w/w NaOH (2.41 mL, 24 mmol, wt.% NaOH of total solution = 4.5) was added and the mixture was stirred at 45 ˚C for 1 h. 1,3-Propane sultone (2.9 g, 24 mmol) was dissolved in acetone (2.3 mL) and added to the mixture. This was stirred for 6 h at 45 ˚C and then at RT overnight (16 h). The suspension was poured into methanol (60 mL). The suspension was filtered with suction and washed with MeOH before drying in a desiccator under vacuum at RT overnight. A white solid, 3.161 g, was recovered.

IR v_{max}/cm^{-1}: 3391br (O-H), 2882 (C-H), 1642 (O-H), 1160 (C-O-C), 1036 (C-O-C), (Spectrum S2).

To test for water-soluble fractions, the filtrate was dried *via* rotary evaporation to leave a white solid. This material was analysed by ^1H NMR and IR. No cellulose was present.

This procedure was identical for:

1,4-butane sultone modified cellulose

(Cellulose mass used = 2.000 g, recovered cellulose mass = 2.633 g).

IR v_{max}/cm^{-1}: 3350br (O-H), 2881 (C-H), 1640 (O-H), 1155 (C-O-C), 1022 (C-O-C), (Spectrum S3).

S2.3 Reaction of cellulose with cyclic anhydrides

Adapted from de Melo *et al.*[4]

Cellulose (0.10 g) and maleic anhydride (2 g) were added to a 20 mL reaction vial with a stir bar (cellulose 5 wt.% compared to maleic anhydride). A vacuum adapter was added to the vial which was vacuum filled with nitrogen (x3) and nitrogen flow was maintained throughout the reaction. The vial was placed in an aluminium block pre-heated to 120 °C. The maleic anhydride melted immediately to form a suspension. The reaction was continued for 6 h. After 6 h the vial was cooled under N_2. Water (ca. 20 mL) was added to dissolve the unreacted maleic anhydride and precipitate the cellulose. The mixture wad filtered and washed with water and acetone before drying overnight under vacuum in a desiccator. A white solid, 0.078 g, was obtained.

IR v_{max}/cm^{-1}: 3335 br (O-H), 1719 (C=O), 1161 (C-O-C), 1106 (C-O-C), 1055 (C-O-C), 1031 (C-O-C), (Spectrum S4).

To test for water-soluble fractions, the filtrate was dried *via* rotary evaporation to leave a white solid. This material was analysed by ^1H NMR and IR. No cellulose was present.

An identical procedure was used to modify cellulose using:

Succinic anhydride

(Cellulose mass used = 0.107 g, recovered cellulose mass = 0.88 g).

IR v_{max}/cm^{-1}: 3336 br (O-H), 1728 (C=O), 1315 (C-H), 1160 (C-O-C), 1107 (C-O-C), 1055 (C-O-C), 1031 (C-O-C), (Spectrum S5).

S2.4 Preparation of water-soluble cellulose

Cellulose previously modified with propane sultone (Section S2.2, 0.10 g) maleic anhydride (2g) were placed in a 20 mL reaction vial with a stir bar (cellulose 5 wt.% compared to maleic anhydride). A

vacuum adapter was added to the vial which was vacuum filled with nitrogen (x3) and nitrogen flow was maintained throughout the reaction. The vial was placed in an aluminium block pre-heated to 120 °C. The maleic anhydride melted immediately to form a suspension. The reaction was continued for 6 h. After 6 h the vial was cooled under N_2. An off-white solid formed. A 1:2 mixture of acetone and water (ca. 50 mL) was added to dissolve the solid. Using rotary evaporation, acetone was first removed and the aqueous solution concentrated until just before solid began to precipitate. Acetone was added until the water-soluble cellulose precipitated. The cellulose was collected *via* centrifugation for 10 min at 10000 RPM and washed with acetone (3 x 10 mL), centrifuging as before. The obtained white solid was dried overnight under vacuum in a desiccator. A white solid, 0.57 g, was obtained.

IR ν_{max}/cm^{-1}: 3402 br (O-H), 2882 (C-H), 1726 (C=O), 1642 (O-H), 1418 (C-H), 1371 (C-H), 1157 (C-O-C), 1105 (C-O-C), 1033 (C-O-C) (Spectrum S6). ^1H NMR (400 MHz, D_2O) δ_H ppm: 6.49 (br d, 0.3H, Maleate $\underline{H}C=C\underline{H}$), 3.01-4.55 (m, 7H, AGU, Sultone $C\underline{H_2}$), 3.01 (s, 0.6H, Sultone $C\underline{H_2}$), 2.05 (s, 0.6H, Sultone $C\underline{H_2}$), (Spectrum S17). ^{13}C NMR (100 MHz, D_2O) δ_C: 71.6 (AGU), 68.7 (AGU), 47.9 ($O_3S\underline{C}H_2CH_2CH_2$), 24.7 ($O_3SCH_2\underline{C}H_2CH_2$), 24.2 ($O_3SCH_2CH_2\underline{C}H_2$), (Spectrum S18).

This procedure was identical, or used with the altered conditions stated below, for the production of water-soluble cellulose by modification with:

Maleic anhydride for 16 h

(Cellulose mass used = 0.101 g, recovered cellulose mass = 0.60 g).

IR ν_{max}/cm^{-1}: 2921 br (O-H), 1717 (C=O), 1633 (O-H), 1155 (C-O-C), 1036 (C-O-C) (Spectrum S7). ^1H NMR (400 MHz, D_2O) δ_H ppm: 6.30 (br d, 0.2H, Maleate $\underline{H}C=C\underline{H}$), 3.02-4.33 (m, 7H, AGU, Sultone $C\underline{H_2}$), 2.80 (s, 0.7H, Sultone $C\underline{H_2}$), 1.84 (s, 0.6H, Sultone $C\underline{H_2}$) (Spectrum S19). ^{13}C NMR (100 MHz, D_2O) δ_C: 72.4 (AGU), 59.2 (AGU), 47.8 ($O_3S\underline{C}H_2CH_2CH_2$), 24.7 ($O_3SCH_2\underline{C}H_2CH_2$), (Spectrum S20).

3 equivalents of maleic anhydride (based on AGU molecular weight of 162.14 $gmol^{-1}$) for 6 h

(Cellulose mass used = 0.507 g, recovered cellulose mass = 0.177 g).

IR ν_{max}/cm^{-1}: 3339 br (O-H), 2898 (C-H), 1724 (C=O), 1640 (O-H), 1422 (C-H), 1370 (C-H), 1161 (C-O-C), 1108 (C-O-C), 1035 (C-O-C) (Spectrum S8). ^1H NMR (400 MHz, D_2O) δ_H ppm: 6.46 (br d, 0.5H, Maleate $\underline{H}C=C\underline{H}$), 3.20-4.48 (m, 7H, AGU, Sultone $C\underline{H_2}$), 32.95 (s, 0.7H, Sultone $C\underline{H_2}$), 1.99 (s, 0.6H, Sultone $C\underline{H_2}$), (Spectrum S21). ^{13}C NMR (100 MHz, D_2O) δ_C: 131.9 (Maleate $H_2\underline{C}=\underline{C}H_2$), 68.7 (AGU), 47.9 ($O_3S\underline{C}H_2CH_2CH_2$), 30.2 ($HOOC\underline{C}H_2CH_2$), 24.2 ($O_3SCH_2CH_2\underline{C}H_2$), (Spectrum S22).

Succinic anhydride for 6 h

(Cellulose mass used = 0.102 g, recovered cellulose mass = 0.044 g).

IR ν_{max}/cm^{-1}: 3405 br (O-H), 1703 (C=O), 1161 (C-O-C), 1041 (C-O-C) (Spectrum S9). ^1H NMR (400 MHz, D_2O) δ_H ppm: 3.18-4.50 (m, 7H, AGU, Sultone $C\underline{H_2}$), 2.96 (s, 0.8H, Sultone $C\underline{H_2}$), 2.19 (s, 0.3H, succinate $C\underline{H_2}$), 1.99 (s, 0.7H, Sultone $C\underline{H_2}$), (Spectrum S23). ^{13}C NMR (100 MHz, D_2O) δ_C: 77.9 (AGU), 72.8 (AGU), 47.8 ($O_3S\underline{C}H_2CH_2CH_2$), 24.7 ($O_3SCH_2\underline{C}H_2CH_2$), (Spectrum S24).

Succinic anhydride for 16 h

(Cellulose mass used = 0.101 g, recovered cellulose mass = 0.033 g).

IR ν_{max}/cm^{-1}: 3404 br (O-H), 1720 (C=O), 1158 (C-O-C), 1035 (C-O-C) (Spectrum S10). ^1H NMR (400 MHz, D_2O) δ_H ppm: 3.24-4.55 (m, 7H, AGU, Sultone $C\underline{H_2}$), 2.97-3.03 (m, 0.8H, Sultone $C\underline{H_2}$), 2.65 (s,

0.4H, succinate $C\underline{H_2}$), 2.01-2,07 (s, 0.7H, Sultone $C\underline{H_2}$), (Spectrum S25). ^{13}C NMR (100 MHz, D_2O) δ_C: 177.8 (Succinate C=O), 74.8 (AGU), 73.7 (AGU), 71.6 (AGU), 69.4 (AGU), 68.7 (AGU), 60.1, (AGU), 47.9 ($O_3S\underline{C}H_2CH_2CH_2$), 29.5 ($HOOC\underline{C}H_2CH_2$), 26.9 ($HOOCCH_2\underline{C}H_2$), 24.8 ($O_3SCH_2\underline{C}H_2CH_2$), 24.2 ($O_3SCH_2CH_2\underline{C}H_2$), (Spectrum S26).

3 equivalents of succinic anhydride (based on AGU molecular weight of 162.14 $gmol^{-1}$) for 6 h

(Cellulose mass used = 0.413 g, cellulose mass recovered = 0.312 g).

IR v_{max}/cm^{-1}: 3419 br (O-H), 1730 (C=O), 1160 (C-O-C), 1040 (C-O-C) (Spectrum S11). ^1H NMR (400 MHz, D_2O) δ_H ppm: 3.24-4.55 (m, 7H, AGU, Sultone $C\underline{H_2}$), 3.01 (m, 0.7H, Sultone $C\underline{H_2}$), 2.76 (s, 0.3H, succinate $C\underline{H_2}$), 2.05(s, 0.8H, Sultone $C\underline{H_2}$), (Spectrum S27). ^{13}C NMR (100 MHz, D_2O) δ_C: 47.8 ($O_3S\underline{C}H_2CH_2CH_2$), 30.2 ($HOOC\underline{C}H_2CH_2$), 28.8 ($HOOCCH_2\underline{C}H_2$), 24.8 ($O_3SCH_2\underline{C}H_2CH_2$), (Spectrum S28).

S2.5 NaOH test reactions

In a 20 mL reaction vial, cellulose (0.67g, 4.13 mmol, RMM assumed to be 162.14 $gmol^{-1}$ based on AGU) was suspended in *i*-PrOH (5 mL) and water (0.6 mL). Then 30 w/w NaOH (0.81 mL, 24 mmol, wt.% NaOH of total solution = 4.5) was added and stirred at 45 ˚C for 1 h. Maleic anhydride (0.82 g, 8.36 mmol) was dissolved in acetone (0.77 mL) and added to the mixture. This was stirred for 6 h at 45 ˚C and then at RT overnight (16 h). The suspension was poured into methanol (15 mL). The suspension was filtered with suction and washed with MeOH before drying in a desiccator under vacuum at RT overnight. A white solid, 0.994 g, was recovered.

IR v_{max}/cm^{-1}: 3312 br (O-H), 2882 (C-H), 1717 (C=O), 1579 (O-H), 1338 (C-H), 1158 (C-O-C), 1021 (C-O-C), (Spectrum S12).

To test for water-soluble fractions, the filtrate was dried *via* rotary evaporation to leave a white solid. This material was analysed by ^1H NMR and IR. No cellulose was present.

This modified cellulose was reacted with maleic anhydride using the procedure below:

Modified cellulose (0.10 g) and maleic anhydride (2 g) were added to a 20 mL reaction vial with a stir bar (cellulose 5 wt.% compared to maleic anhydride). A vacuum adapter was added to the vial which was vacuum filled with nitrogen (x3) and nitrogen flow was maintained throughout the reaction. The vial was placed in an aluminium block pre-heated to 120 °C. The maleic anhydride melted immediately to form a suspension. The reaction was continued for 6 h. After 6 h the vial was cooled under N_2. Water (ca. 20 mL) was added to dissolve the unreacted maleic anhydride and precipitate the cellulose. The mixture wad filtered and washed with water and acetone before drying overnight under vacuum in a desiccator. 0.098 g solid was obtained.

IR v_{max}/cm^{-1}: 2920 (C-H), 1725 (C=O), 1635 (O-H), 1403 (C-H), 1158 (C-O-C), 1033 (C-O-C), (Spectrum S13).

To test for water-soluble fractions, the filtrate was dried *via* rotary evaporation to leave a white solid. This material was analysed by ^1H NMR and IR. No cellulose was present.

This procedure was identical for the modification of cellulose using:

Succinic anhydride

NaOH reaction: (Cellulose mass used = 0.67 g, recovered cellulose mass = 1.35 g).

IR v_{max}/cm^{-1}: 3419 br (O-H), 2883 (C-H), 1714 (C=O), 1573 (O-H), 1157 (C-O-C), 1021 (C-O-C), (Spectrum S14).

Subsequent succinic anhydride reaction: (Modified cellulose mass used = 0.10 g, recovered cellulose mass = 0.03 g).

IR v_{max}/cm^{-1}: 2919 (C-H), 1712 (C=O), 1570 (O-H), 1357 (C-H), 1150 (C-O-C), 1046 (C-O-C), (Spectrum S15).

S2.6 Preparation of a fluorescent cellulose derivative

Cellulose previously modified with propane sultone (Section S2.2, 0.20 g), maleic anhydride (1g), and 1,8-naphthalic anhydride (1 g) were placed in a 20 mL reaction vial with a stir bar (cellulose 10 wt.% compared to total anhydride). A vacuum adapter was added to the vial which was vacuum filled with nitrogen (x3) and nitrogen flow was maintained throughout the reaction. The vial was placed in an aluminium block pre-heated to 120 °C. The maleic anhydride melted immediately to form a suspension of cellulose and 1,8-naphthalic anhydride. The reaction was continued for 16 h. After 16 h the vial was cooled under N_2.

Purification was carried out in one of two ways:

A 1:2 mixture of acetone and water (ca. 50 mL) was added to dissolve the solid. Using rotary evaporation, acetone was first removed and the aqueous solution concentrated until just before solid began to precipitate. Acetone was added until the water-soluble cellulose precipitated. The cellulose was collected *via* centrifugation for 10 min at 10000 RPM and washed with acetone (3 x 10 mL), centrifuging as before. The mixture wad filtered and washed with acetone before drying under vacuum.

Acetone (ca. 30 mL) was added to dissolve unreacted anhydride. The mixture wad filtered and washed with acetone before drying under vacuum.

A slightly water-soluble white solid, was obtained. A suspension in water fluoresced under 365 nm light.

IR v_{max}/cm^{-1}: 3391, br (O-H), 2882 (C-H), 1716 (C=O), 1641 (O-H), 1419 (C-H), 1369 (C-H), 1159 (C-O-C), 1109 (C-O-C), 1040 (C-O-C), (Spectrum S16). ^{1}H NMR (400 MHz, D_2O) δ_H ppm: 6.49 (dd, *J* = 12.2, 2.8 Hz, 0.2H, Maleate $\underline{H}C{=}C\underline{H}$), 5.70 (dd, *J* = 12.2, 2.5 Hz, 0.2H, Maleate $\underline{H}C{=}C\underline{H}$), 3.00-4.45 (m, 7H, AGU, Sultone $C\underline{H_2}$), 2.77-2.91 (m, 1.49H, Sultone $C\underline{H_2}$), 1.83-1.89 (m, 1.33H, Sultone $C\underline{H_2}$), (Spectrum S29). ^{13}C NMR (100 MHz, D_2O) δ_C: 185.0 (Maleate $H_2\underline{C}{=}\underline{C}H_2$), 132.8 (Maleate $H_2\underline{C}{=}\underline{C}H_2$), 68.8 (AGU), 68.6 (AGU), 47.8 ($O_3S\underline{C}H_2CH_2CH_2$), (Spectrum S30).

S3 Degree of Substitution (DS) Calculation

S3.1 Homo-Substituted Cellulose

For homo-substituted cellulose the degree of substitution was calculated from elemental analysis. Unmodified cellulose was analysed and found to have a carbon content of 42.03. Graphs of theoretical carbon content at degree of substitution 1, 2, and 3 were plotted for maleic anhydride (Figure S1) and succinic anhydride (Figure S2). With these graphs, the measured carbon content of the modified cellulose was used to determine the degree of substitution. For 1,3-propane sultone modified cellulose, a similar procedure was used with sulfur content (Figure S3).

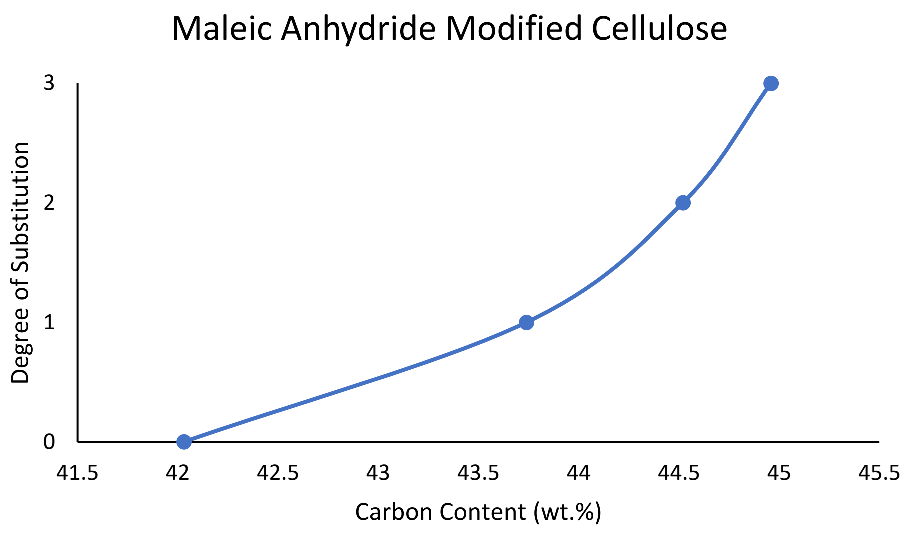

Degree of Substitution	Carbon Content (wt.%)
0	42.03
1	43.74
2	44.52
3	44.96

Figure S1: The graph used to determine the degree of substitution for maleic anhydride modified cellulose.

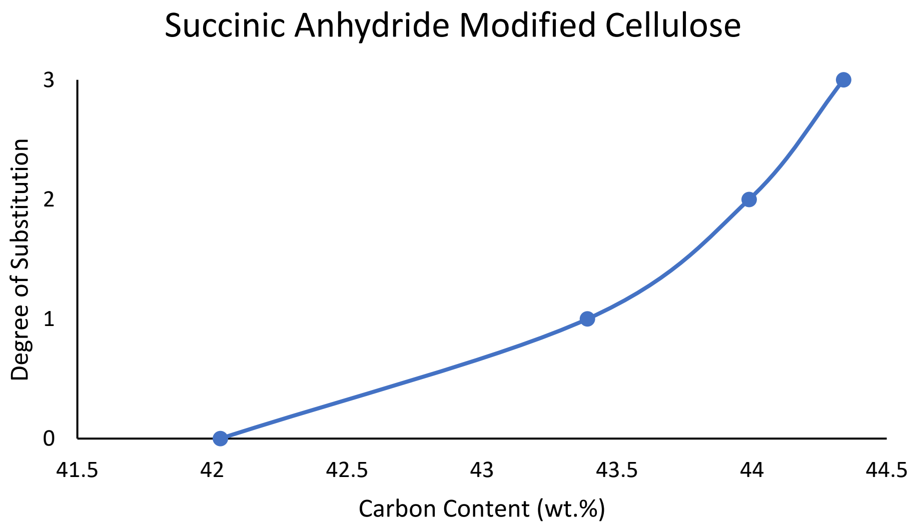

Degree of Substitution	Carbon Content (wt.%)
0	42.03
1	43.19
2	43.89
3	43.55

Figure S2: The graph used to determine the degree of substitution for succinic anhydride modified cellulose.

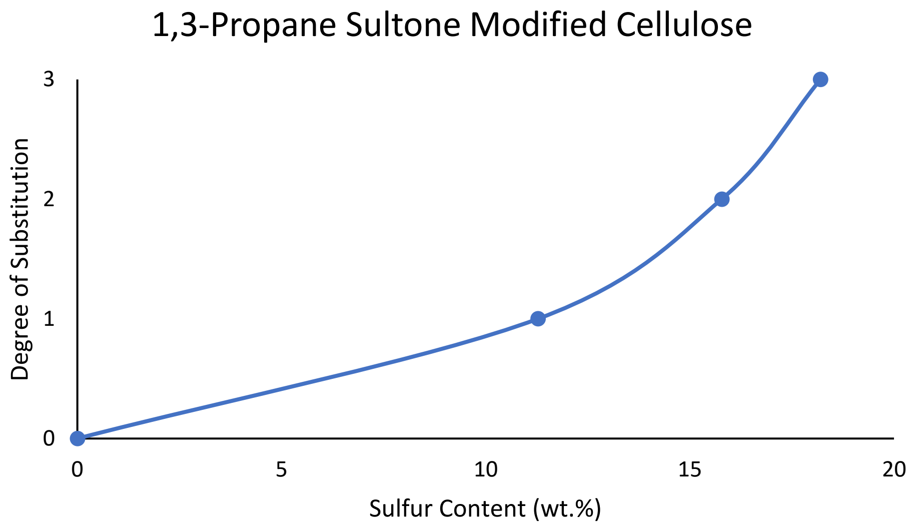

Degree of Substitution	Sulfur Content (wt.%)
0	0.00
1	11.28
2	15.78
3	18.20

Figure S3: The graph used to determine the degree of substitution for 1,3-propane sultone modified cellulose.

S3.2 Hetero-substituted cellulose

Elemental analysis could not be used to calculate the degree of substitution of hetero-substituted cellulose as the theoretical carbon content could not be accurately estimated. Therefore, a modified back-titration method was used to determine the DS of the carboxylate groups. This was added to the DS of the sulfate groups previously determined by elemental analysis, by assuming that the sulfate group existed as the sodium salt.[5,6] A representative procedure and calculation is given below:

0.035 g modified cellulose was suspended in 22 mL 0.009 M NaOH and stirred for 1 h. The pH of the NaOH was measured before adding modified cellulose and after the 1 h reaction. The pH before = 11.45, pH after = 11.08

The moles of carboxylate substituent groups was calculated using equations ia-iiia.

ia) pOH = 14 – pH

iia) $[OH^-] = 10^{-pOH}$

iiia) moles NaOH = (concentration x volume) / 1000

The moles NaOH was therefore calculated to be = 3.56×10^{-5}

The degree of substitution was calculated using equations ib-vb

ib) Moles carboxylate substituents = change of moles NaOH = 3.56×10^{-5}

iib) Moles anhydroglucose unit = mass modified cellulose / molecular weight anhydroglucose unit = 0.035 / 162.14 = 2.15×10^{-4}

iiib) % Carboxylate substitution = moles substituent groups / moles anhydroglucose unit = $3.56 \times 10^{-5} / 2.15 \times 10^{-4} = 0.17$

ivb) DS Carboxylate = % Substitution x Maximum DS = 0.17 x 3 = 0.51

vb) Total DS = DS Carboxylate + DS Sulfate = 0.51 + 0.60 = 1.11

S4 Infrared (IR) Spectra

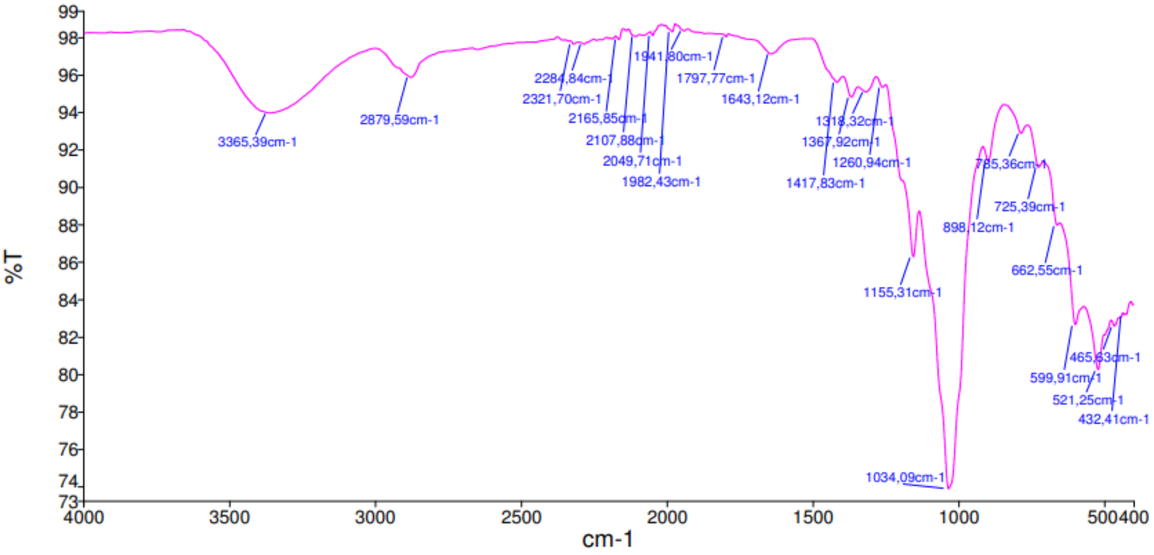

Spectrum S1: IR spectrum of NaOH treated cellulose (Section S2.1).

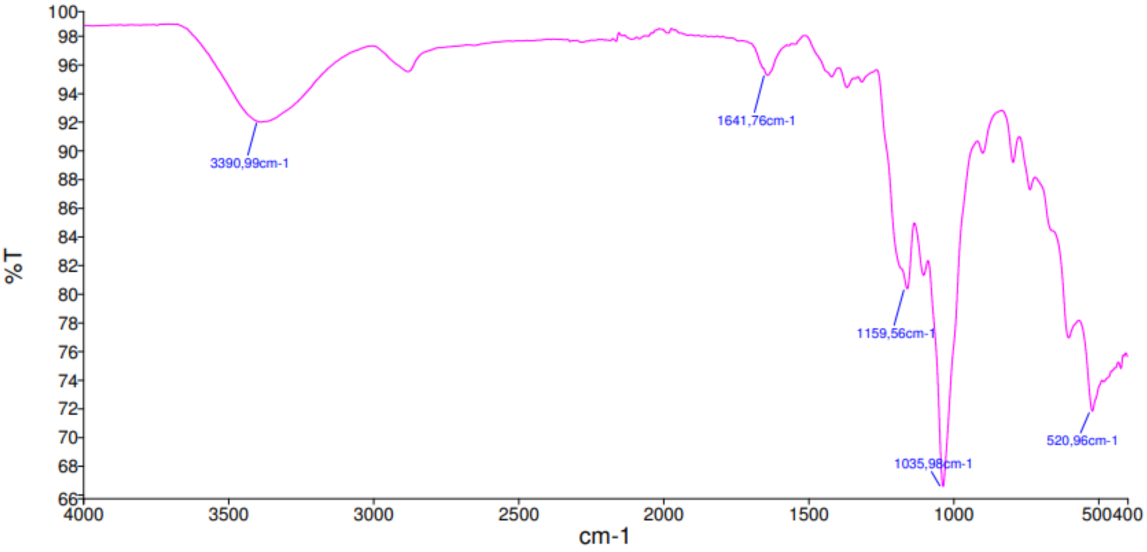

Spectrum S2: IR spectrum of cellulose reacted with 1,3-propane sultone (Section S2.2).

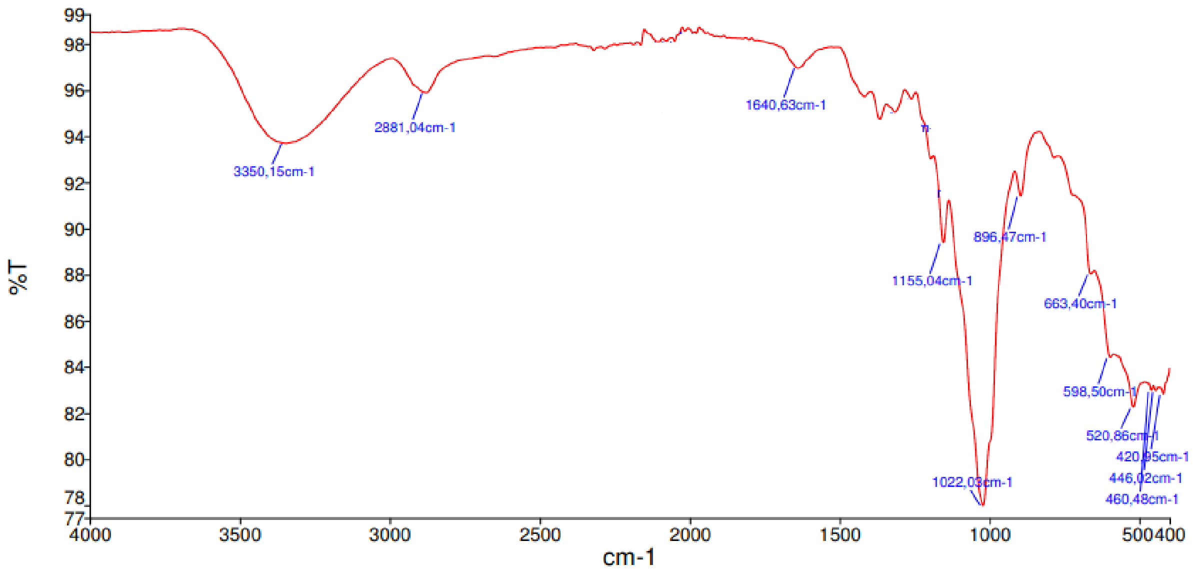

Spectrum S3: IR spectrum of cellulose reacted with 1,4-butane sultone (Section S2.2).

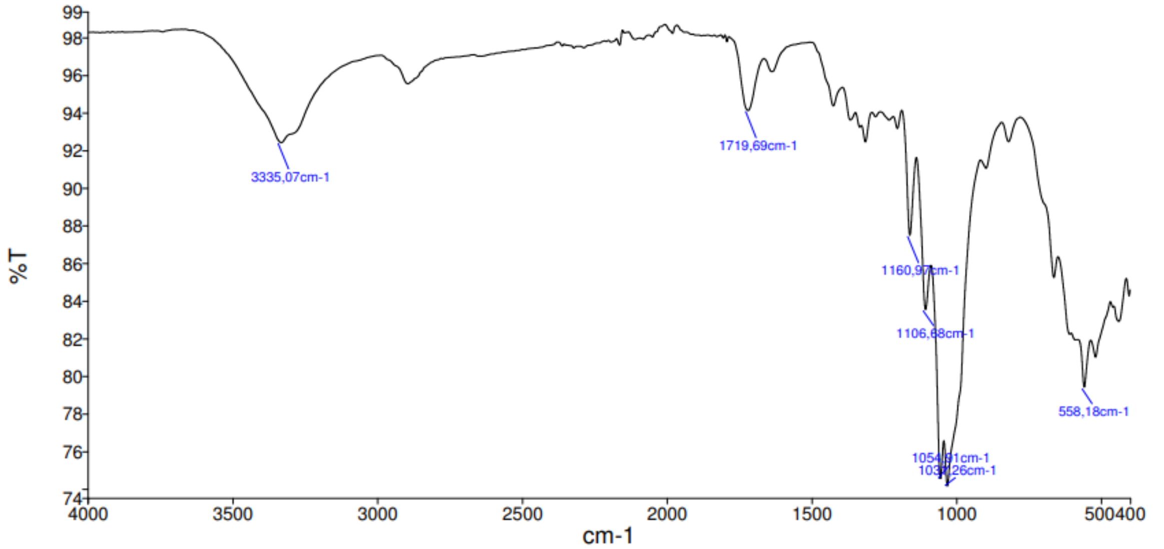

Spectrum S4: IR spectrum of cellulose reaction with maleic anhydride (Section S2.3).

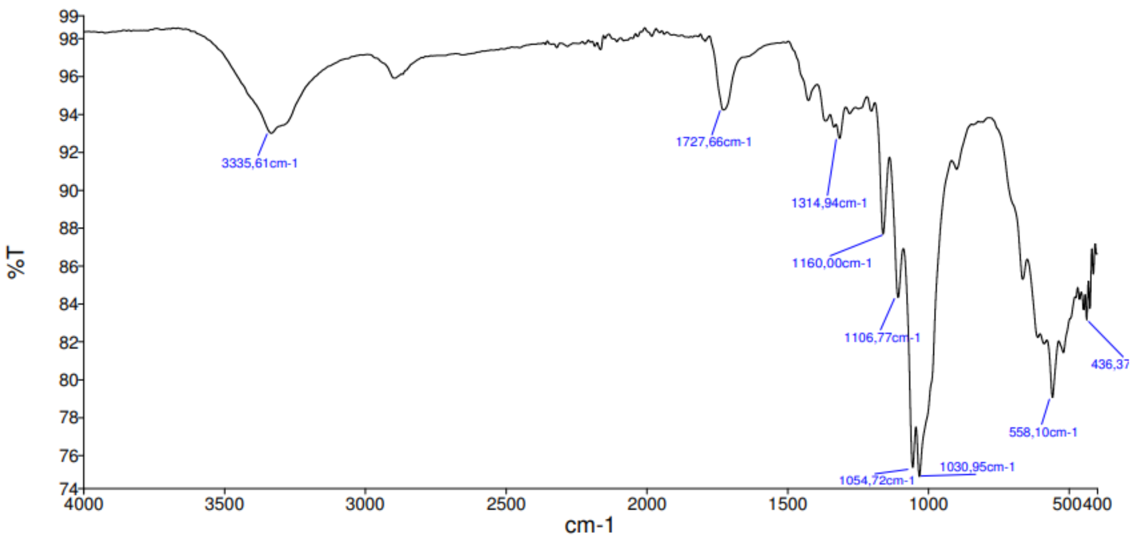

Spectrum S5: IR spectrum of cellulose reaction with succinic anhydride (Section S2.3).

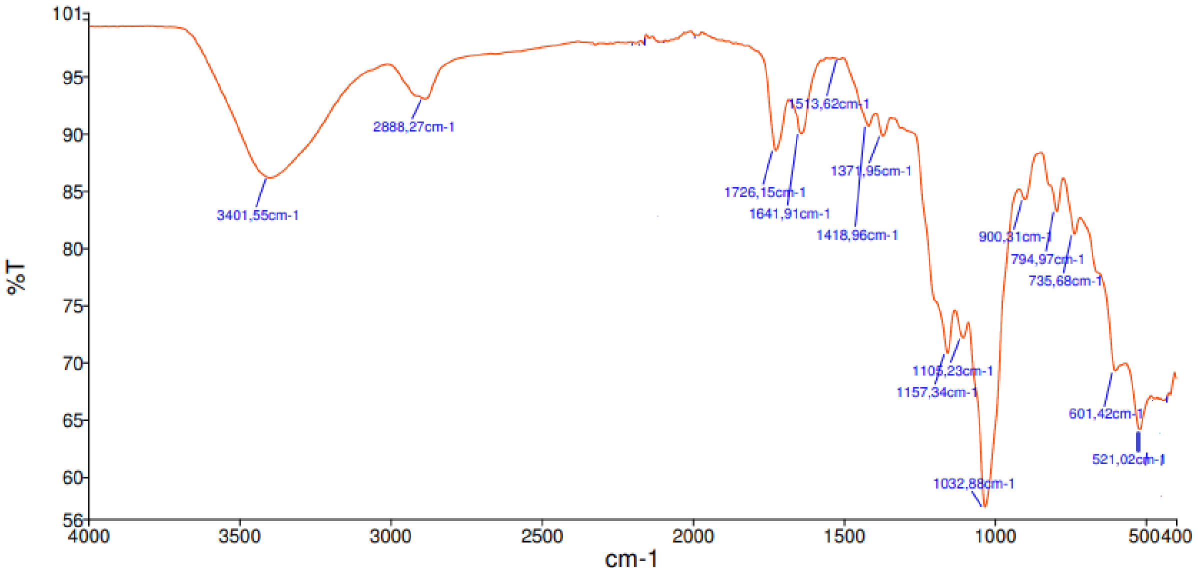

Spectrum S6: IR spectrum of water-soluble cellulose prepared using a 6 h reaction with maleic anhydride (Section S2.4).

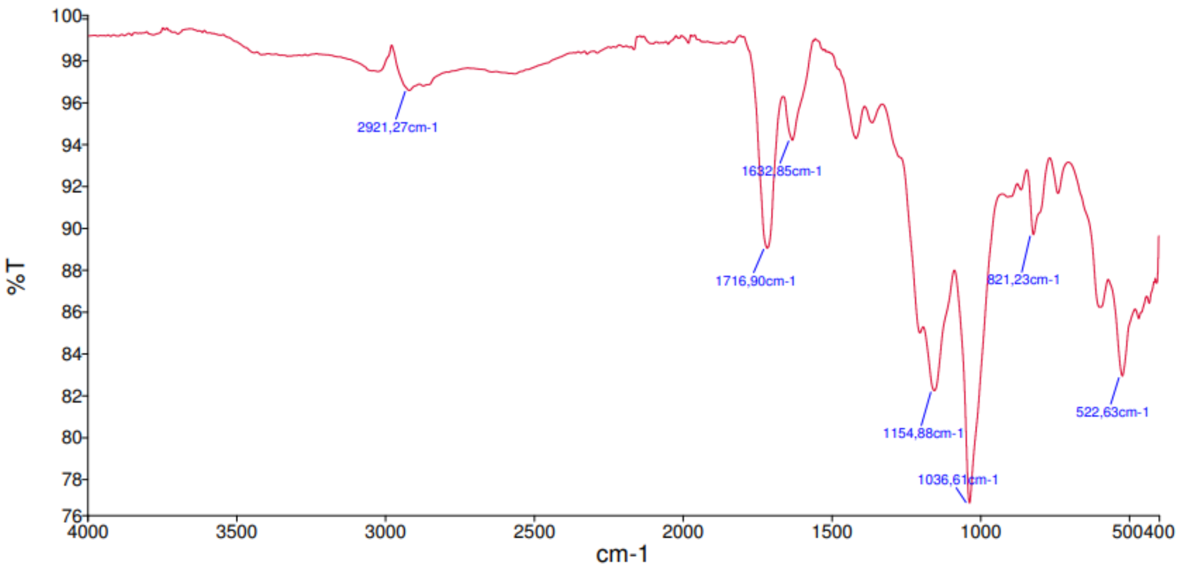

Spectrum S7: IR spectrum of water-soluble cellulose prepared using a 16 h reaction with maleic anhydride (Section S2.4).

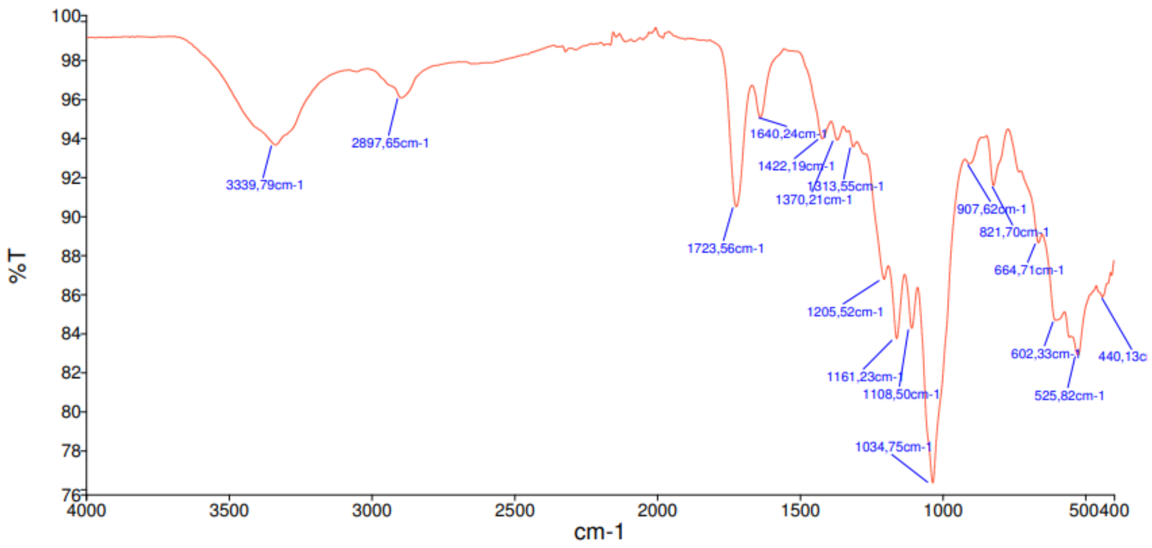

Spectrum S8: IR spectrum of water-soluble cellulose prepared by a reaction with 3 equiv. maleic anhydride (Section S2.4).

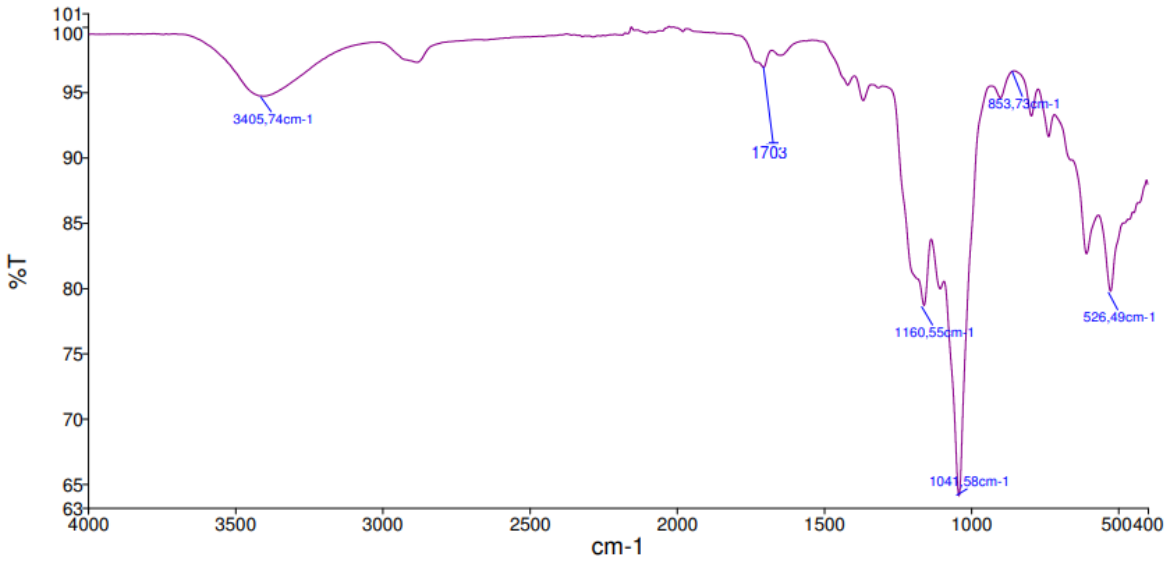

Spectrum S9: IR spectrum of water-soluble cellulose prepared using a 6 h reaction with succinic anhydride (Section S2.4).

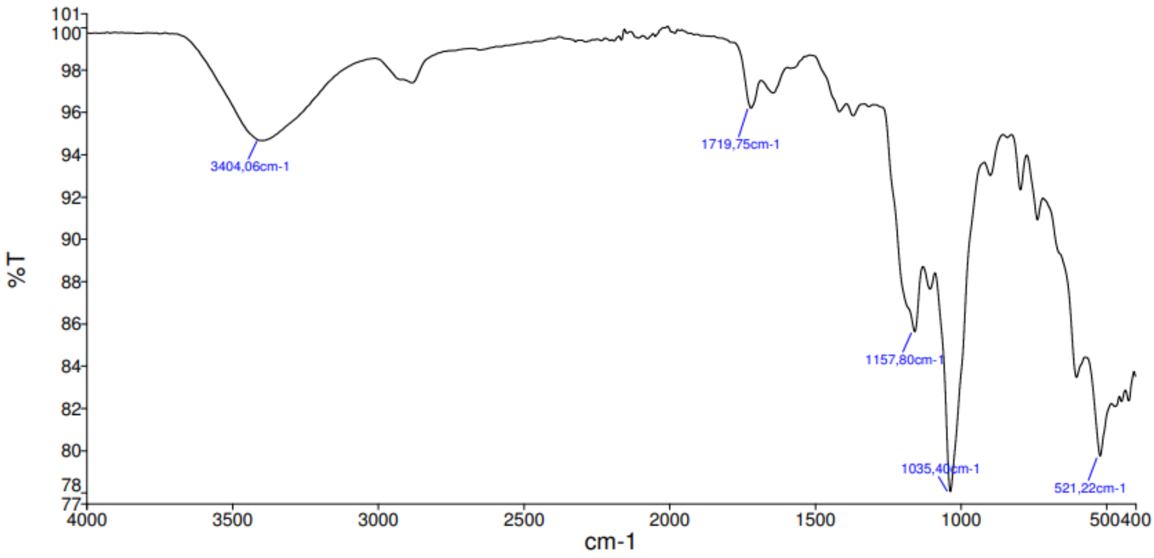

Spectrum S10: IR spectrum of water-soluble cellulose prepared using a 16 h reaction with succinic anhydride (Section S2.4).

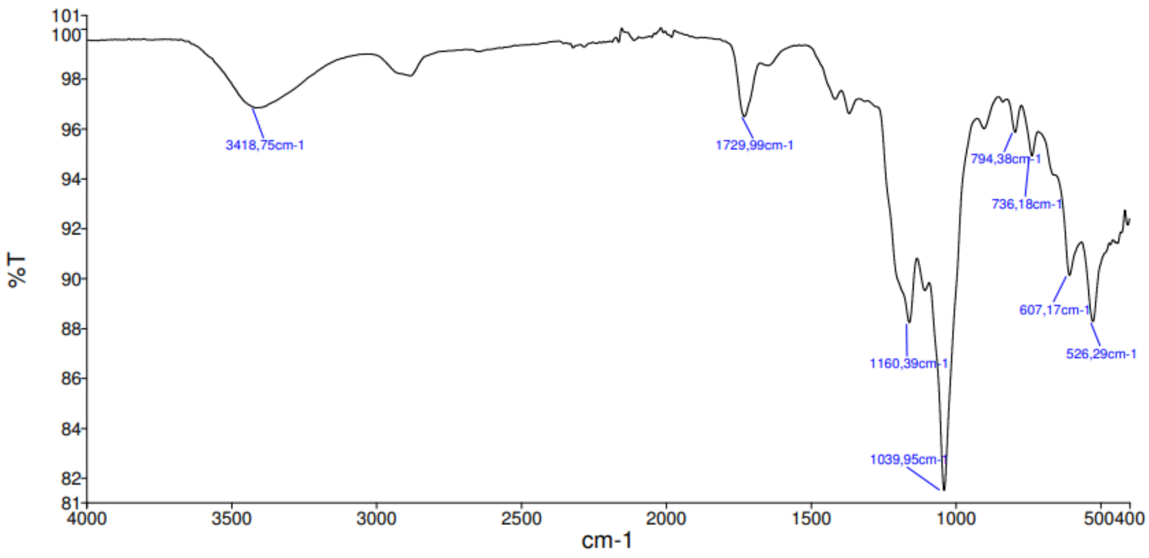

Spectrum S11: IR spectrum of water-soluble cellulose prepared by a reaction with 3 equiv. succinic anhydride (Section S2.4).

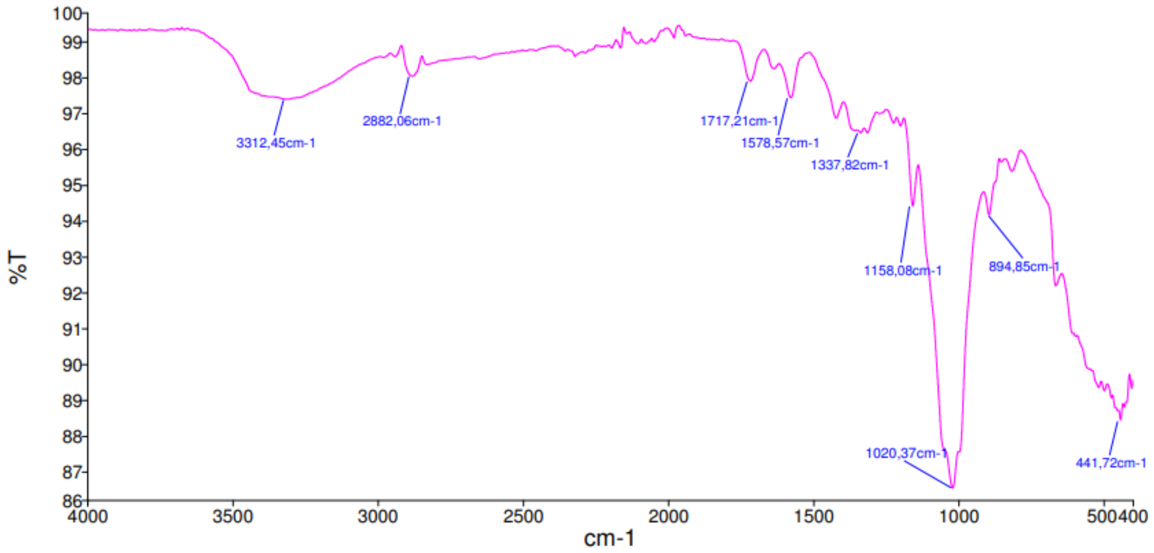

Spectrum S12: IR spectrum of NaOH test reaction of cellulose with maleic anhydride (Section S2.5).

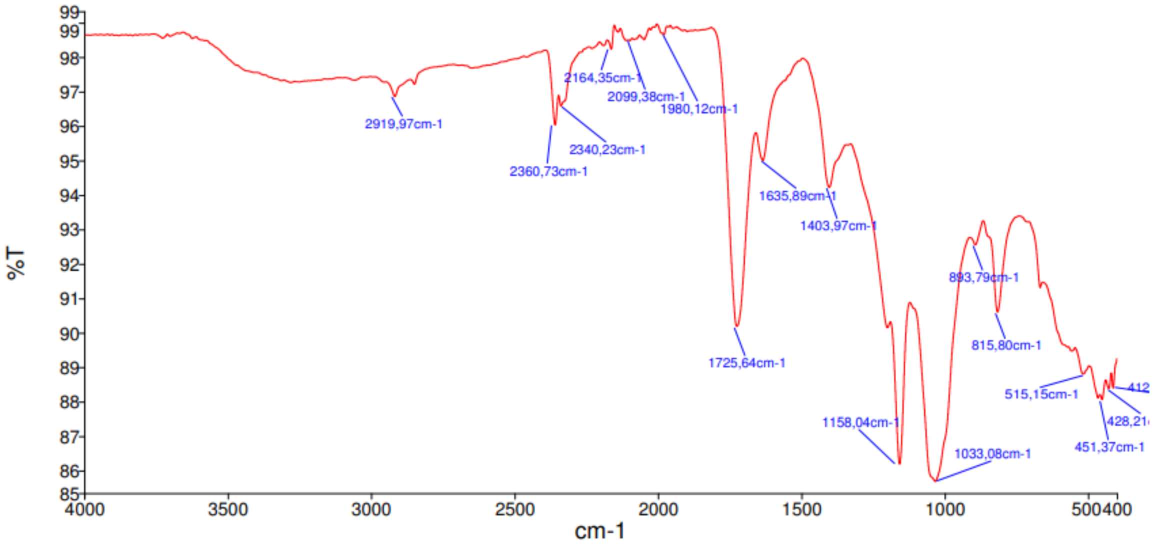

Spectrum S13: IR spectrum of NaOH test reaction of cellulose with maleic anhydride, followed by subsequent reaction with maleic anhydride (Section S2.5).

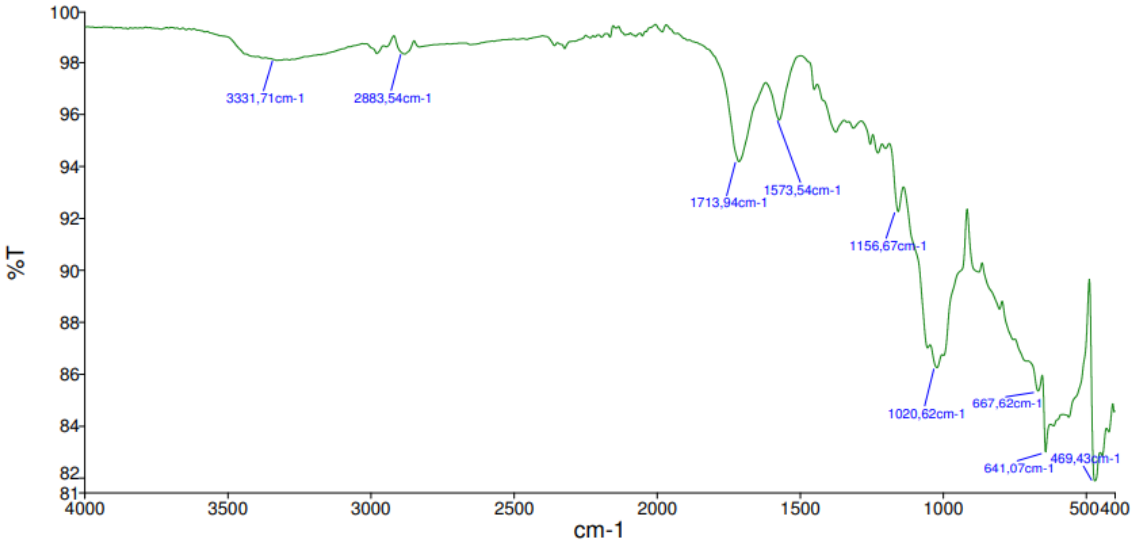

Spectrum S14: IR spectrum of NaOH test reaction of cellulose with succinic anhydride (Section S2.5).

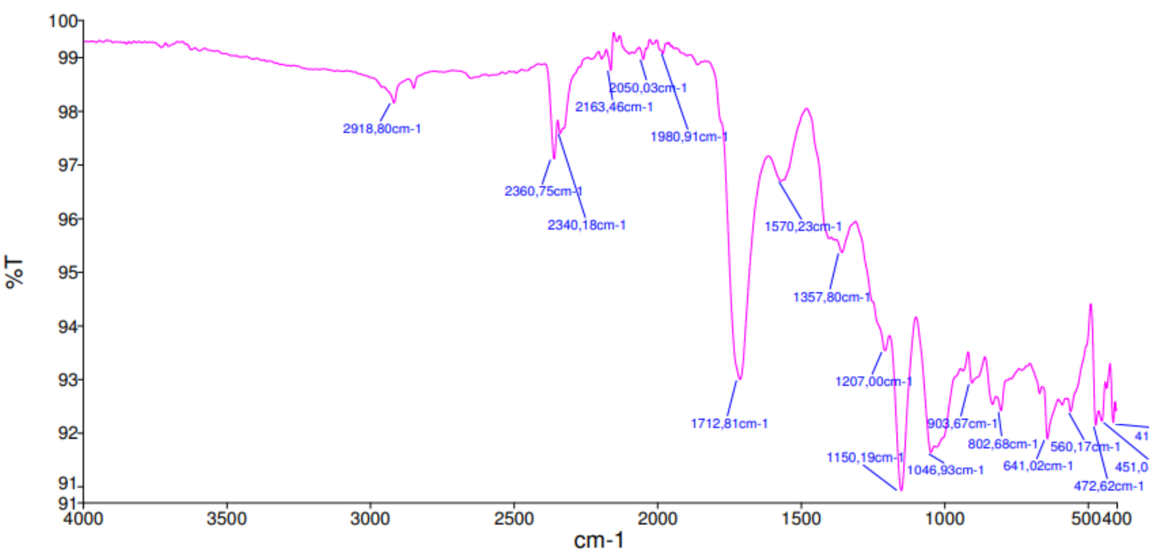

Spectrum S15: IR spectrum of NaOH test reaction of cellulose with succinic anhydride followed by subsequent reaction with succinic anhydride (Section S2.5).

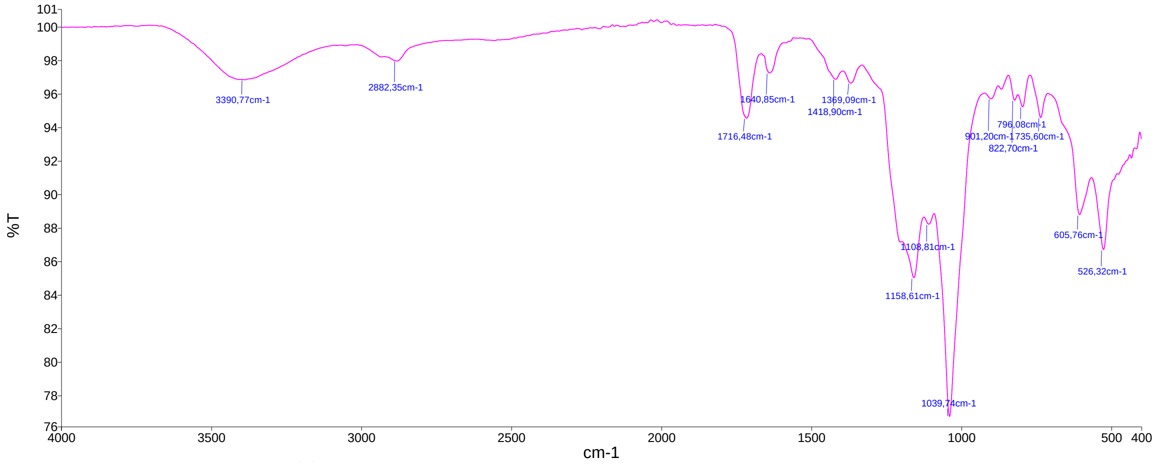

Spectrum S16: IR spectrum of fluorescent cellulose prepared by a reaction with maleic anhydride and 1,8-naphthalic anhydride (Section S2.6).

S5 Nuclear Magnetic Resonance (NMR) spectra

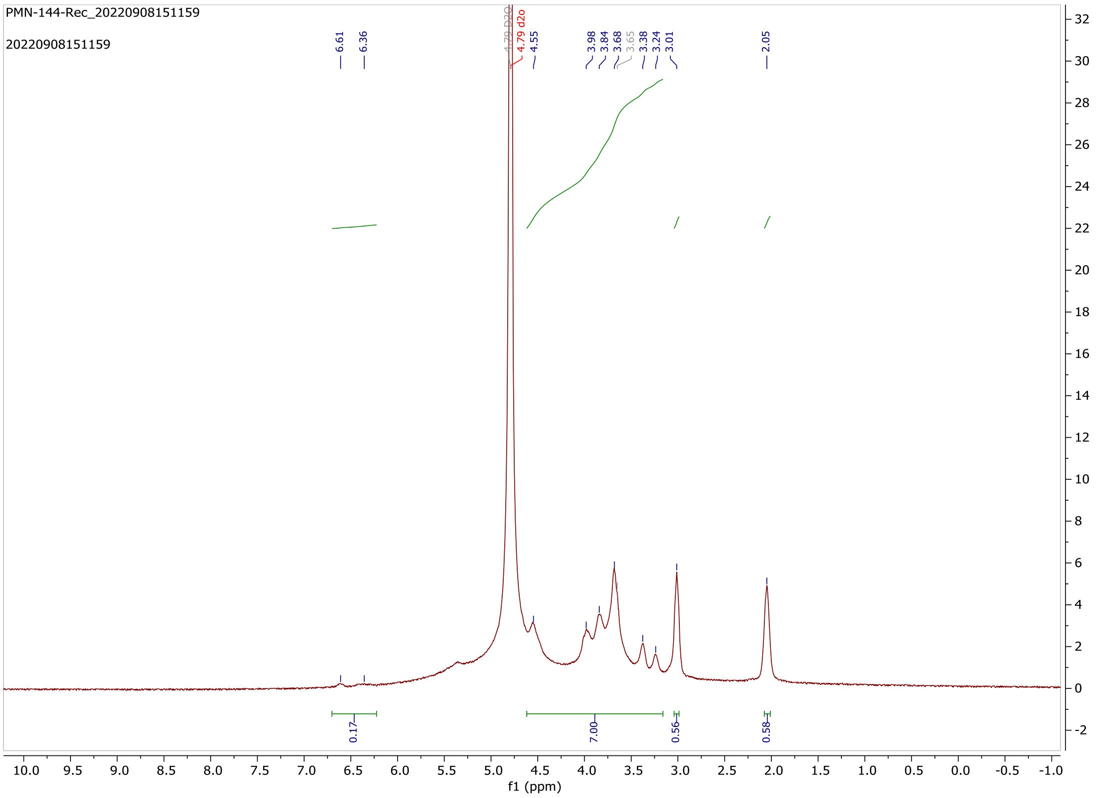

Spectrum S17: ^{1}H NMR (D_2O, 400 MHz) spectrum of water-soluble cellulose prepared using a 6 h reaction with maleic anhydride (Section S2.4).

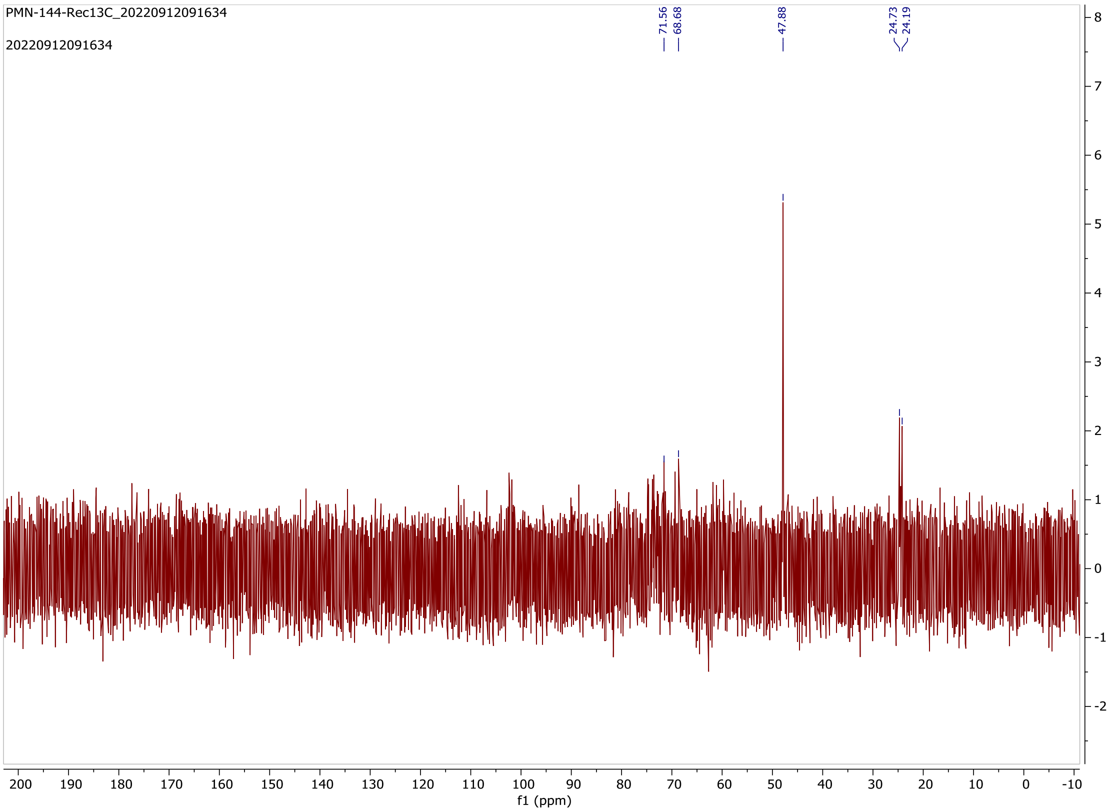

Spectrum S18: ^{13}C NMR (D_2O, 100 MHz) spectrum of water-soluble cellulose prepared using a 6 h reaction with maleic anhydride (Section S2.4).

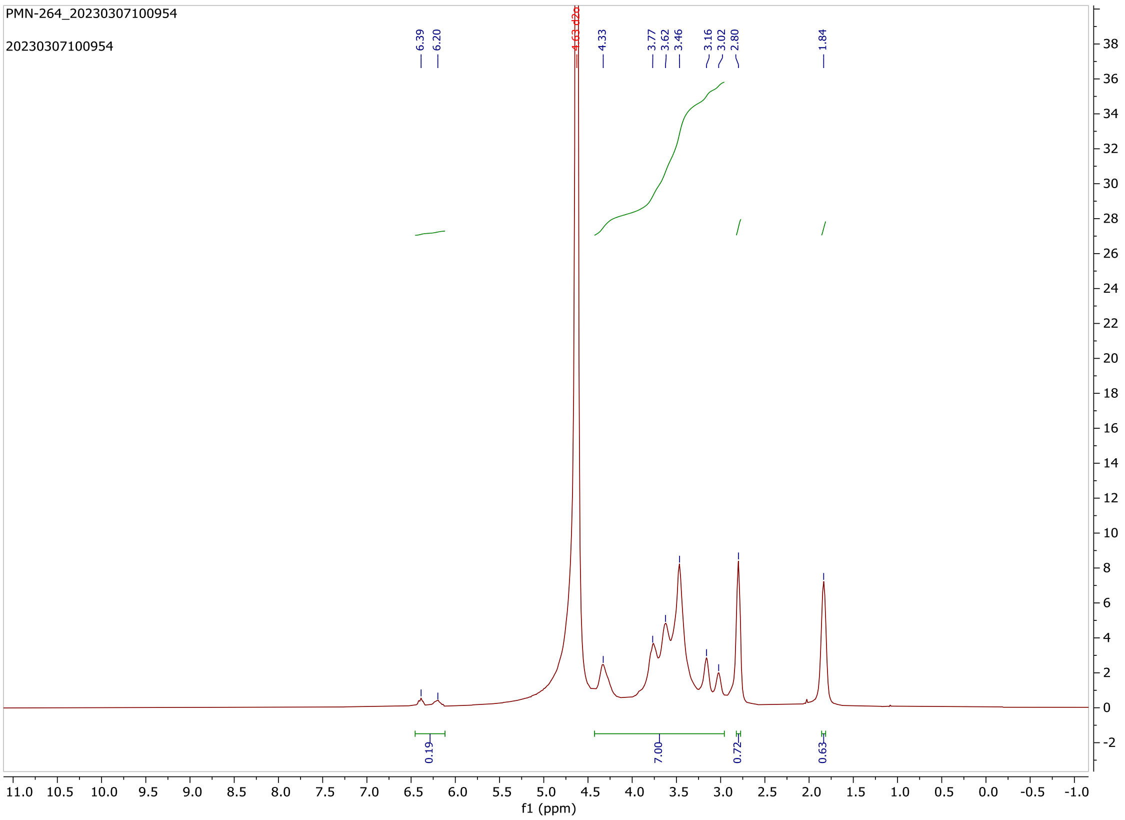

Spectrum S19: ^{1}H NMR (D_2O, 400 MHz) spectrum of water-soluble cellulose prepared using a 16 h reaction with maleic anhydride (Section S2.4).

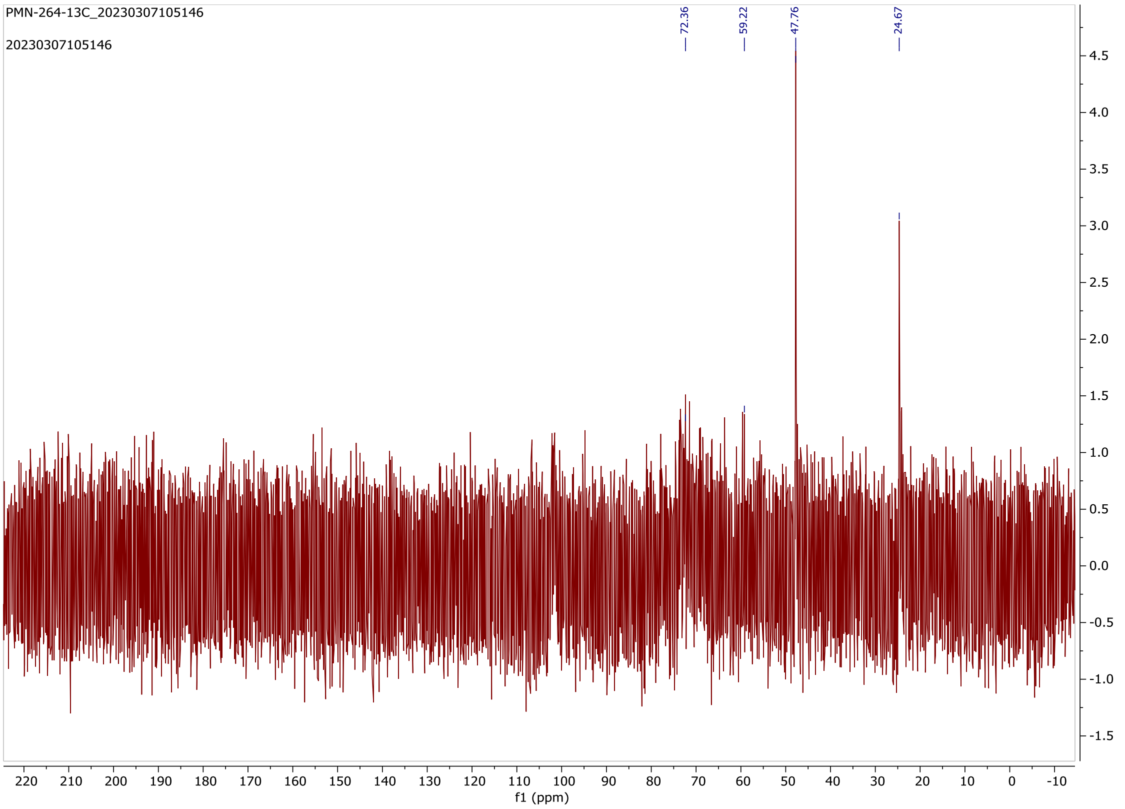

Spectrum S20: ^{13}C NMR (D_2O, 100 MHz) spectrum of water-soluble cellulose prepared using a 16 h reaction with maleic anhydride (Section S2.4).

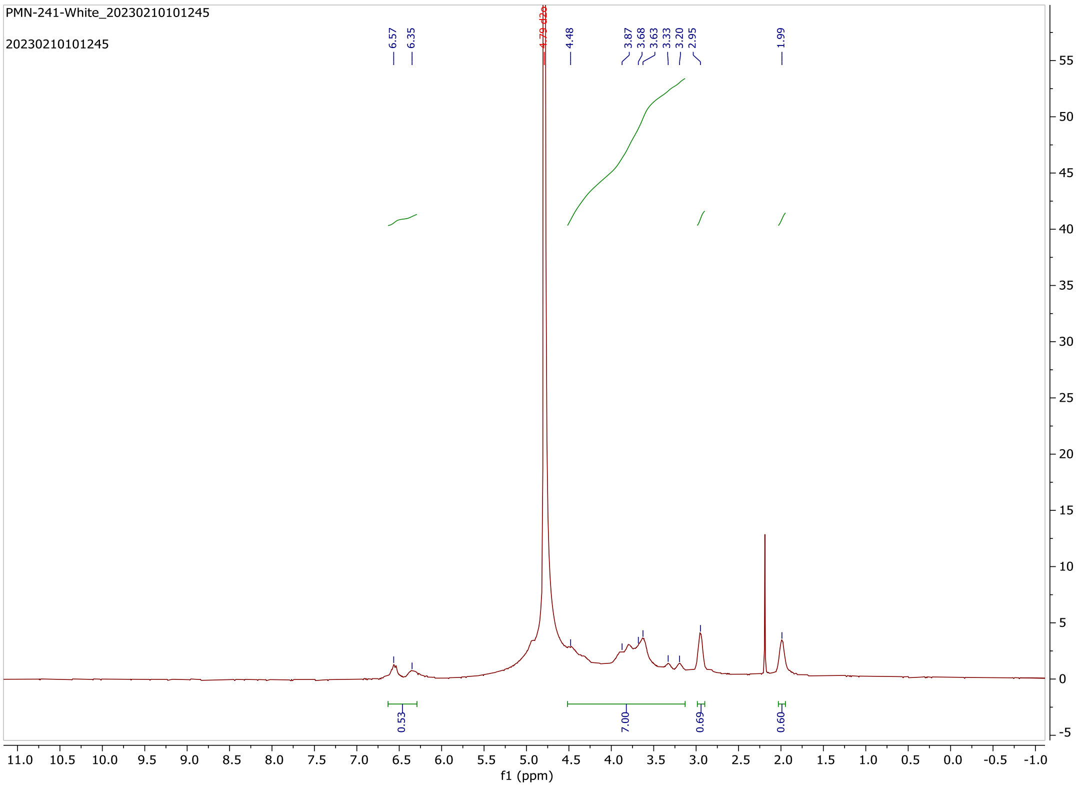

Spectrum S21: ^{1}H NMR (D_2O, 400 MHz) spectrum of water-soluble cellulose prepared by a reaction with 3 equiv. maleic anhydride (Section S2.4).

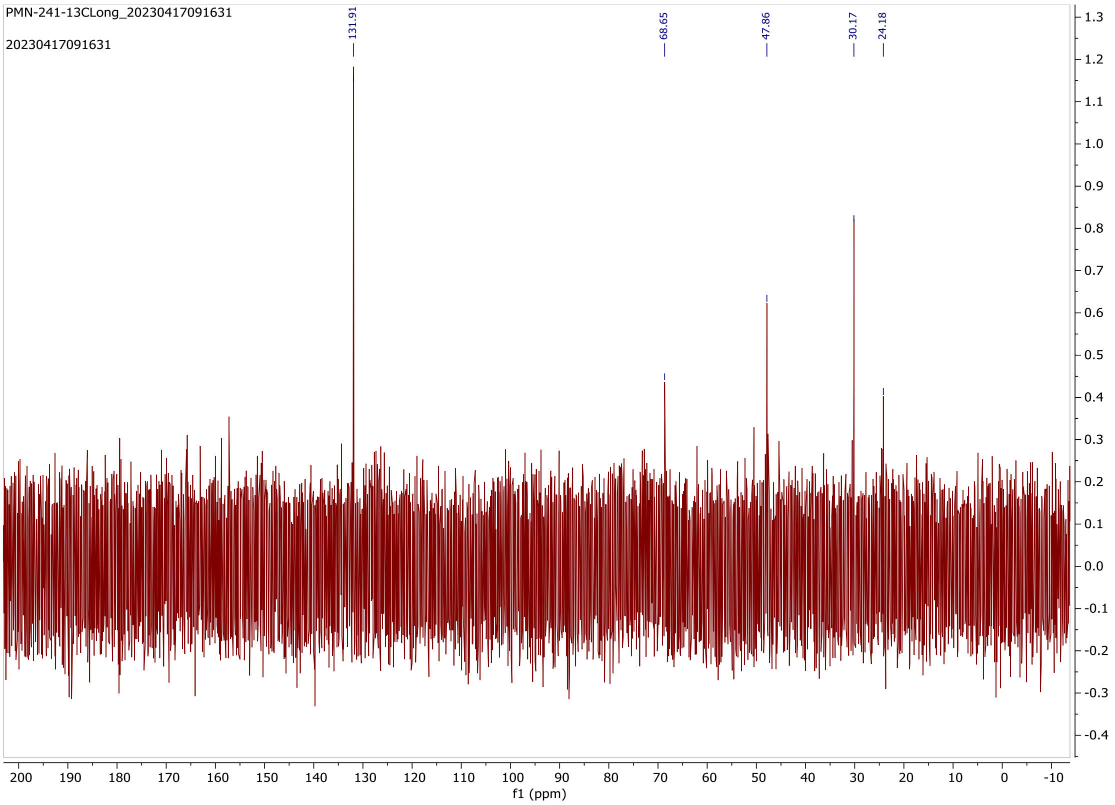

Spectrum S22: ^{13}C NMR (D_2O, 100 MHz) spectrum of water-soluble cellulose prepared by a reaction with 3 equiv. maleic anhydride (Section S2.4).

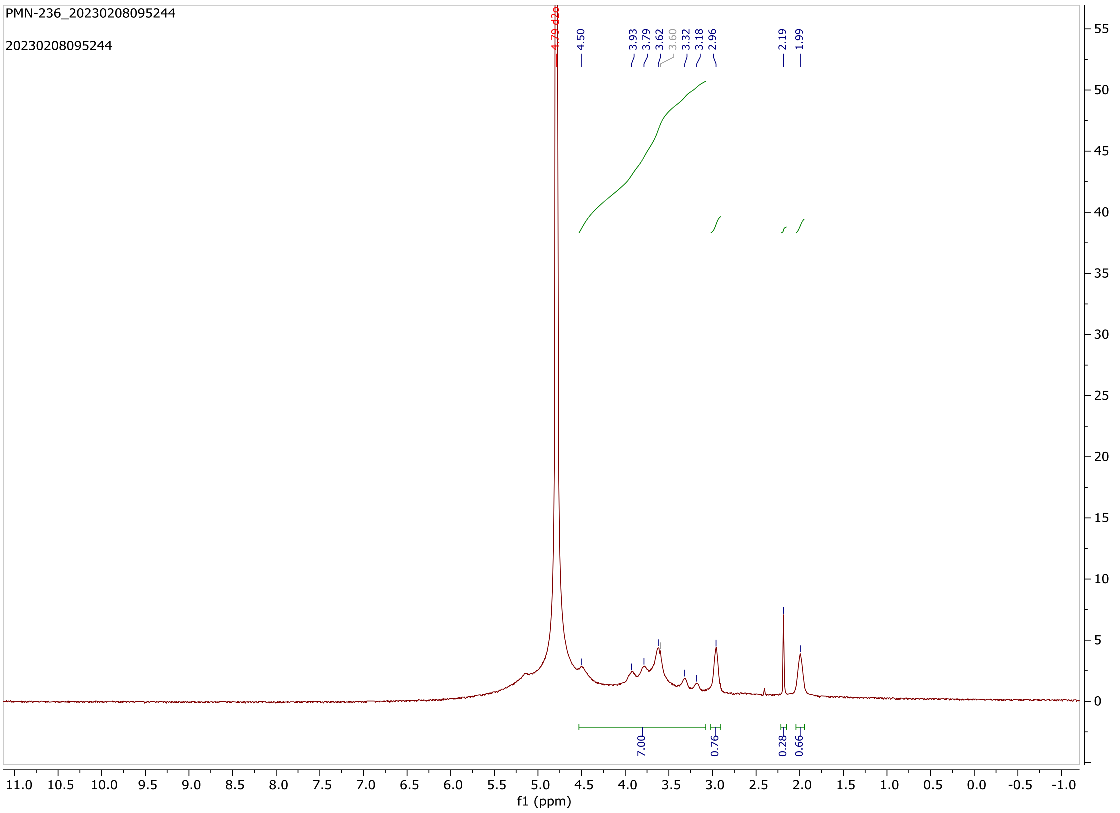

Spectrum S23: ^{1}H NMR (D_2O, 400 MHz) spectrum of water-soluble cellulose prepared using a 6 h reaction with succinic anhydride (Section S2.4).

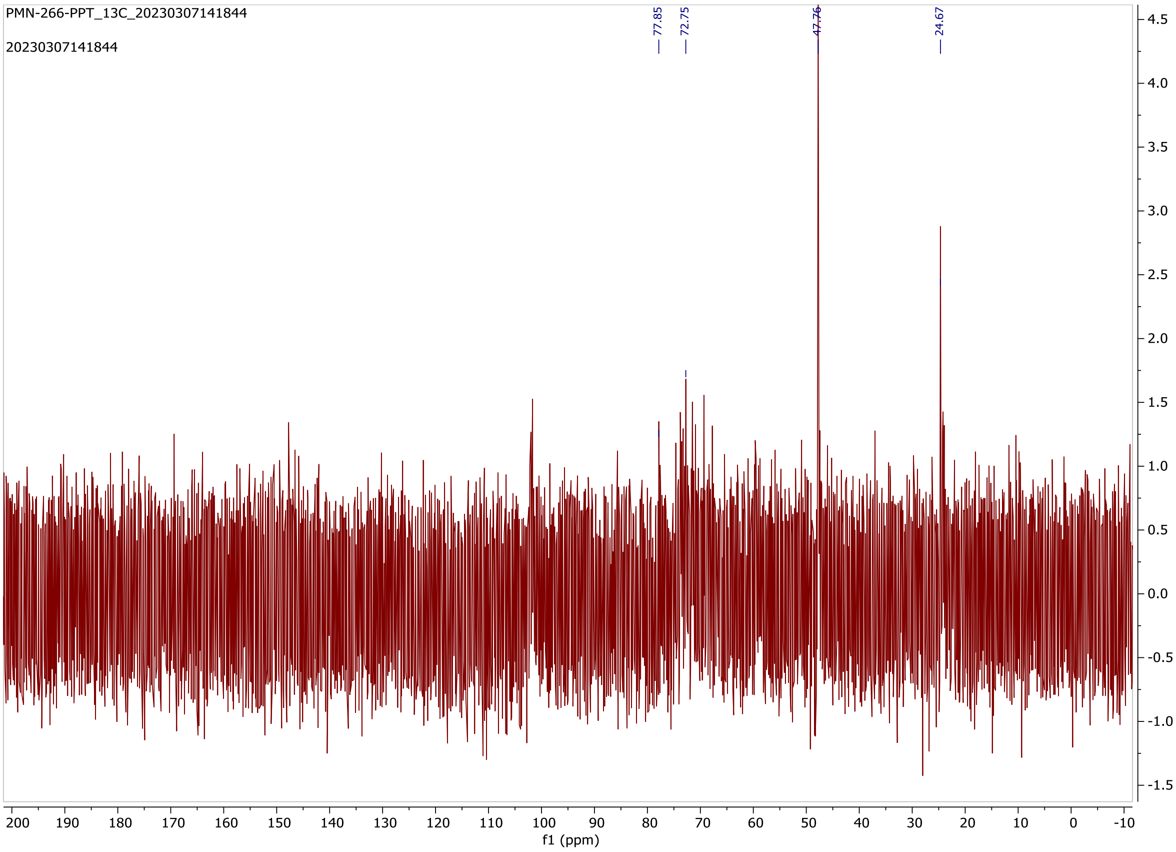

Spectrum S24: ^{13}C NMR (D_2O, 100 MHz) spectrum of water-soluble cellulose prepared using a 6 h reaction with succinic anhydride (Section S2.4).

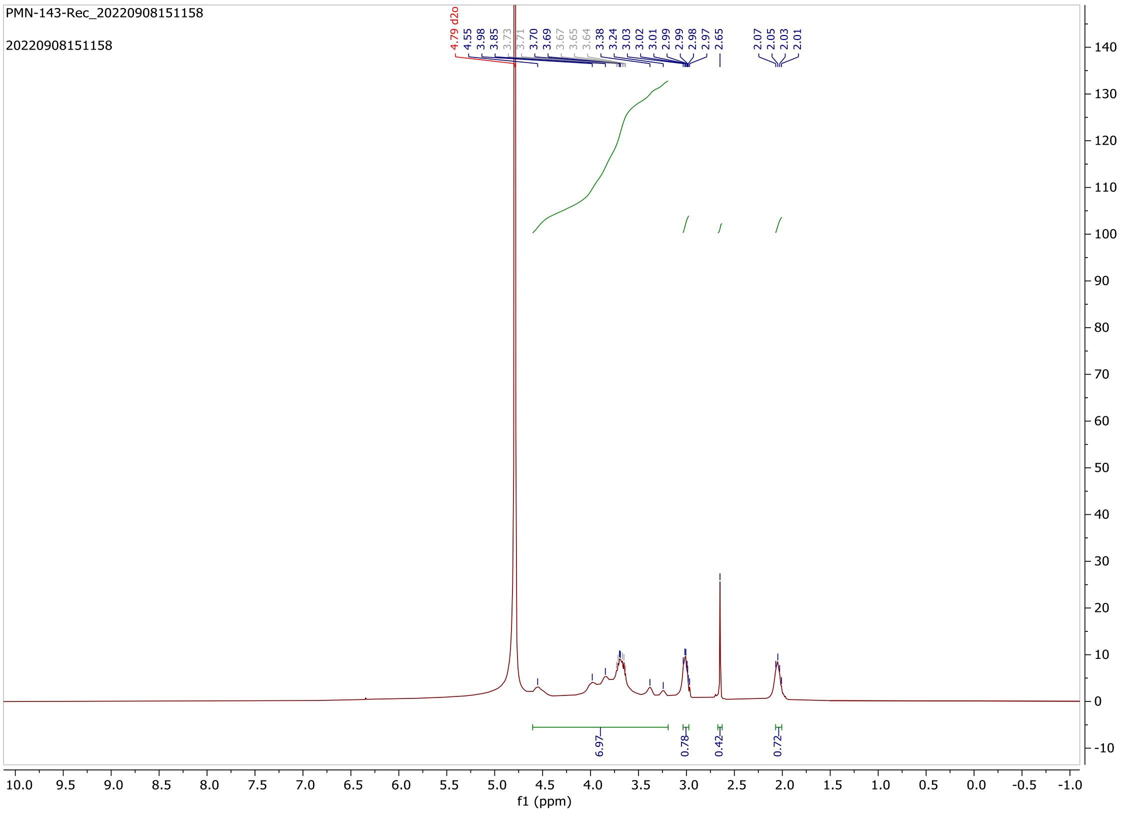

Spectrum S25: ^{1}H NMR (D_2O, 400 MHz) spectrum of water-soluble cellulose prepared using a 16 h reaction with succinic anhydride (Section S2.4).

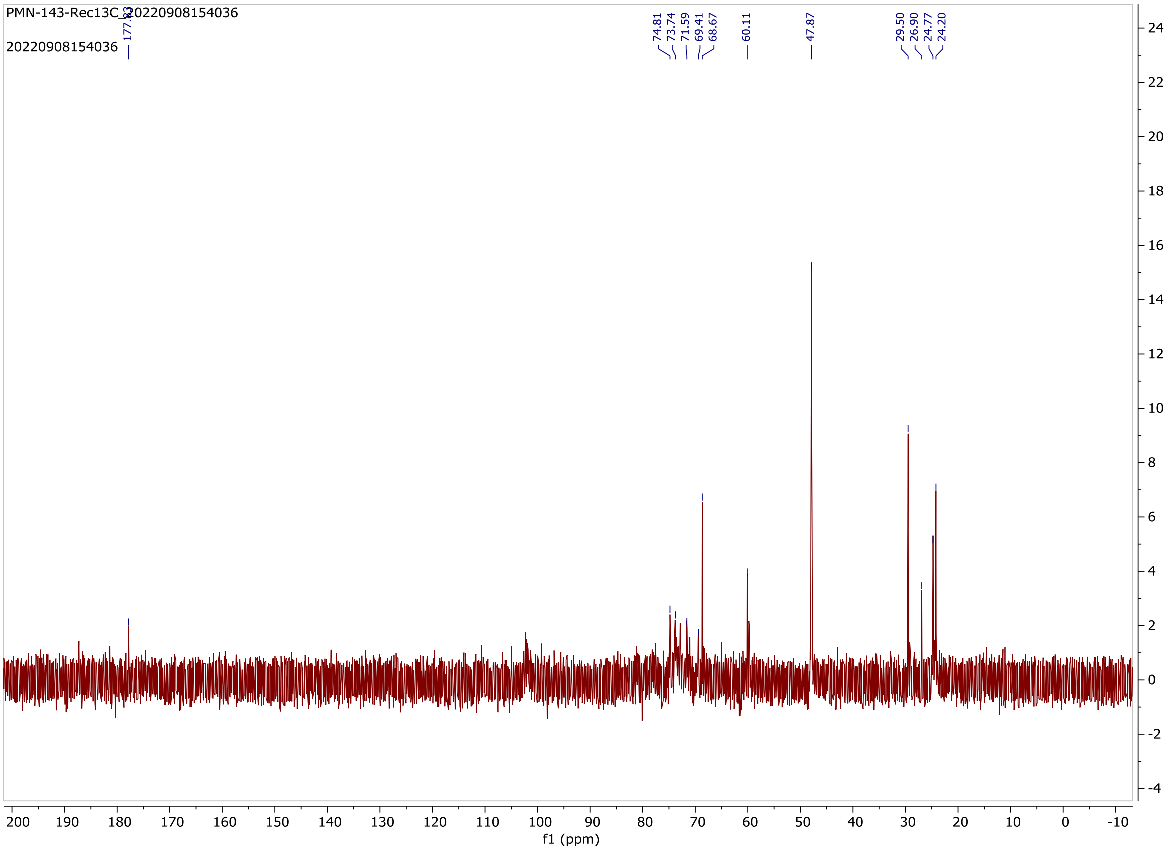

Spectrum S26: ^{13}C NMR (D_2O, 100 MHz) spectrum of water-soluble cellulose prepared using a 16 h reaction with succinic anhydride (Section S2.4).

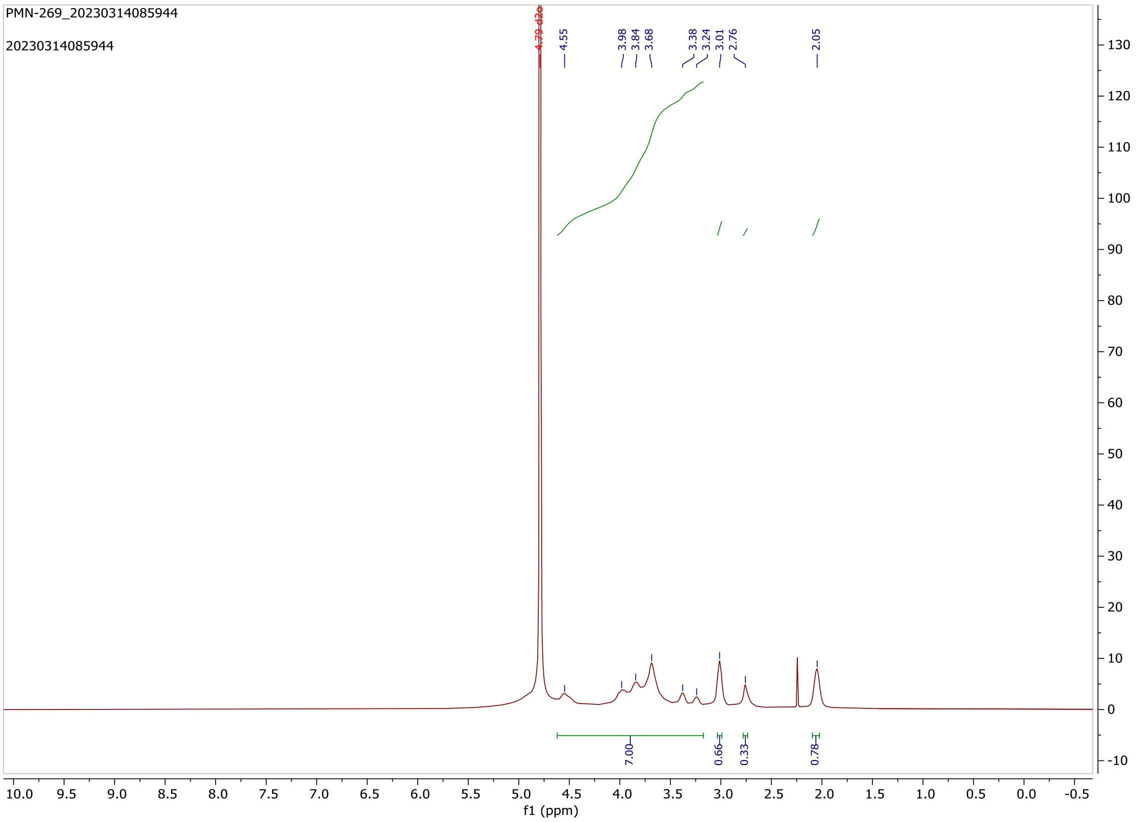

Spectrum S27: ^{1}H NMR (D_2O, 400 MHz) spectrum of water-soluble cellulose prepared by a reaction with 3 equiv. succinic anhydride (Section S2.4).

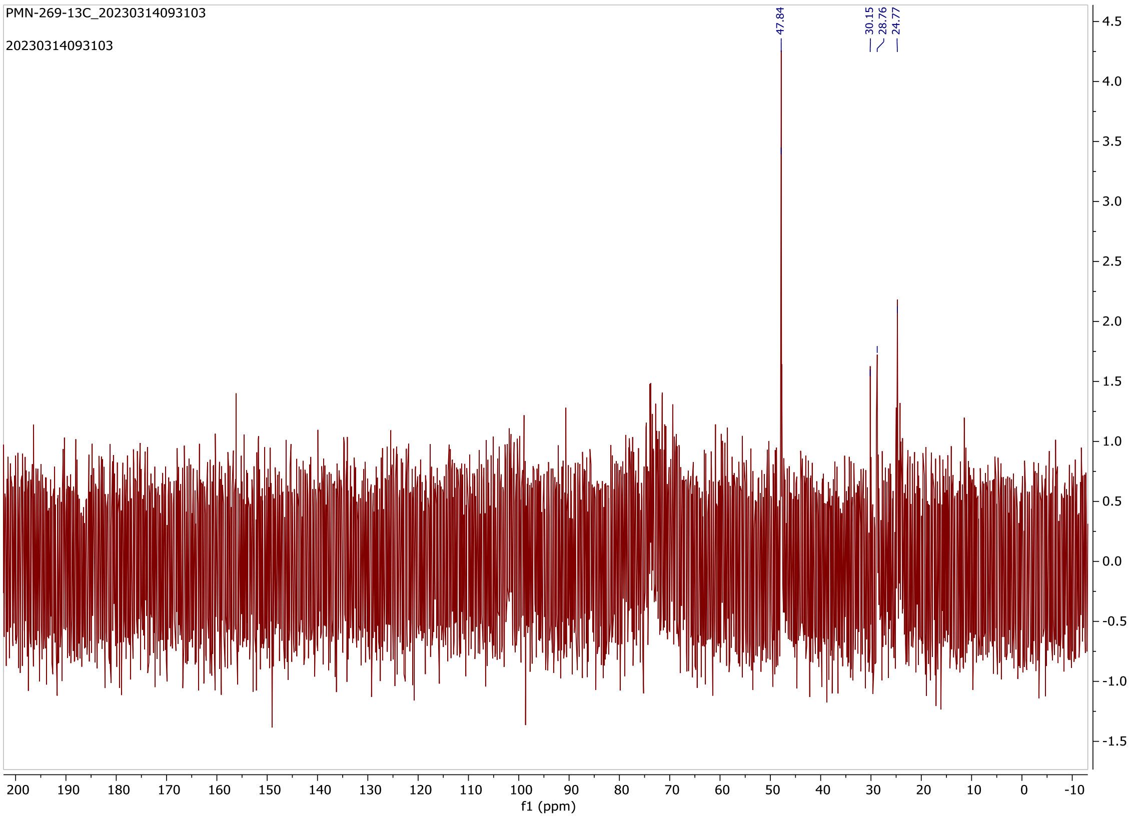

Spectrum S28: ^{13}C NMR (D_2O, 400 MHz) spectrum of water-soluble cellulose prepared by a reaction with 3 equiv. succinic anhydride (Section S2.4).

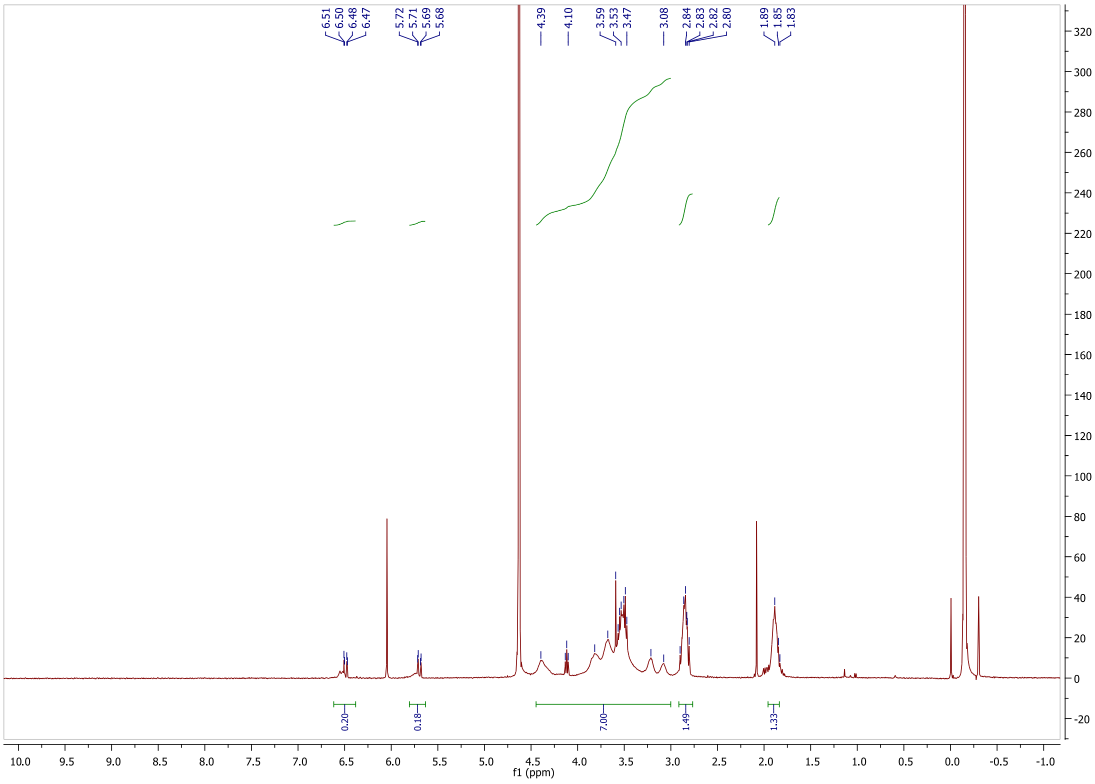

Spectrum S29: ^{1}H NMR (D_2O, 400 MHz) spectrum of fluorescent cellulose prepared by a reaction with maleic anhydride and 1,8-naphthalic anhydride (Section S2.6).

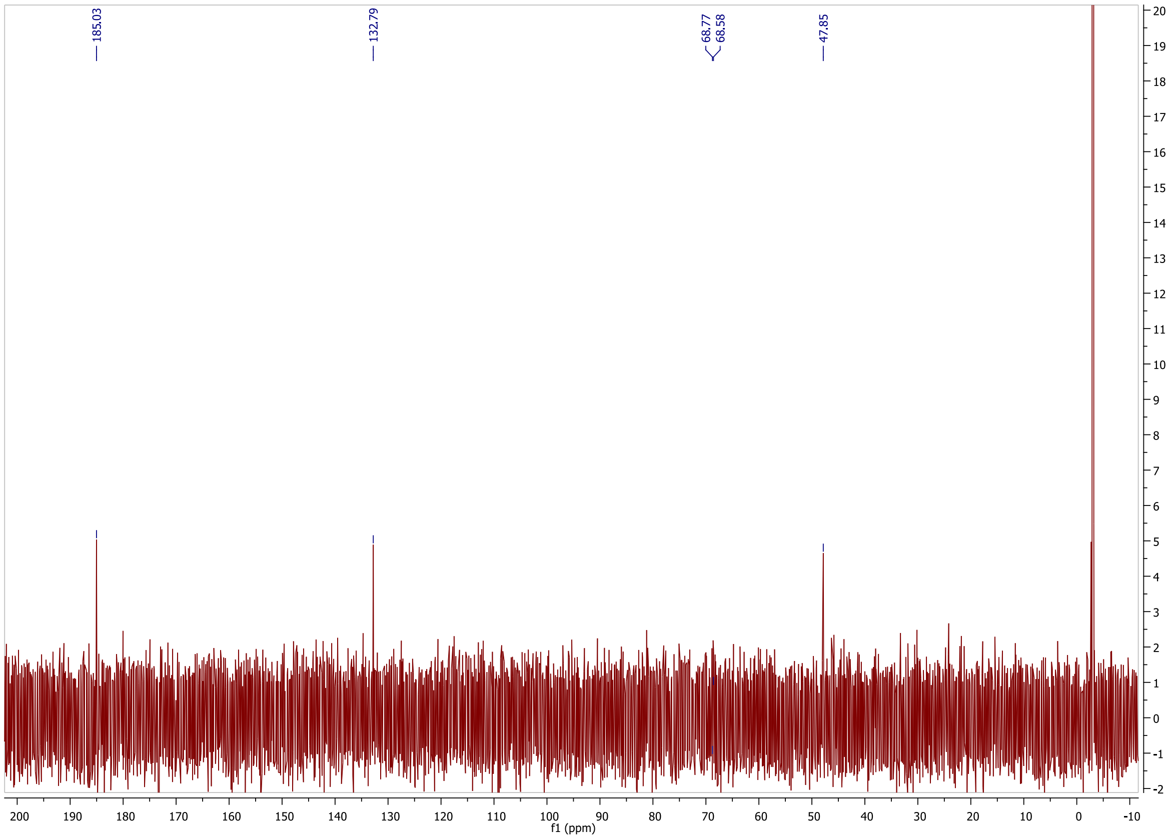

Spectrum S30: ^{13}C NMR (D_2O, 400 MHz) spectrum of fluorescent cellulose prepared by a reaction with maleic anhydride and 1,8-naphthalic anhydride (Section S2.6).

S6 Gel Permeation Chromatography (GPC) Data

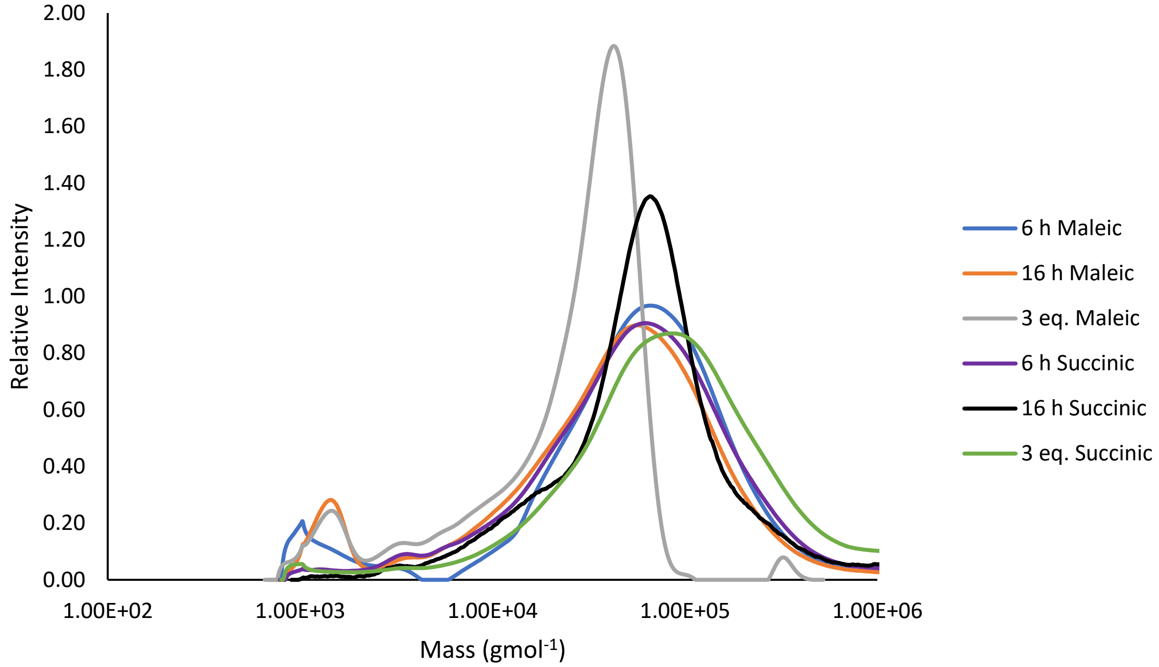

Figure S4: A plot of the GPC traces for water-soluble cellulose.

Table S1: Mw and Mn values of water-soluble cellulose obtained from GPC using a Pullulan calibration.

Entry	Name	Mw ($gmol^{-1}$)	Mn ($gmol^{-1}$)
**1**	6 h Maleic	145900	46970
**2**	16 h Maleic	93830	31970
**3**	3 equiv. Maleic	34550	22980
**4**	6 h Succinic	109400	22710
**5**	16 h Succinic	130700	31910
**6**	3 equiv. Succinic	242500	28410

S7 X-Ray Diffraction (XRD) Analysis

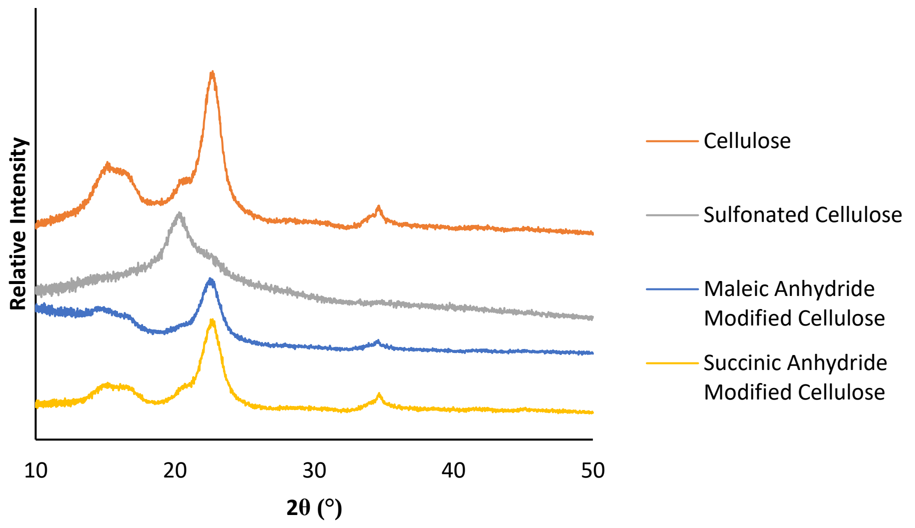

Figure S5: XRD pattern obtained for cellulose, sulfonated cellulose, maleic anhydride modified cellulose and succinic anhydride modified cellulose (Main Paper, Scheme 1).

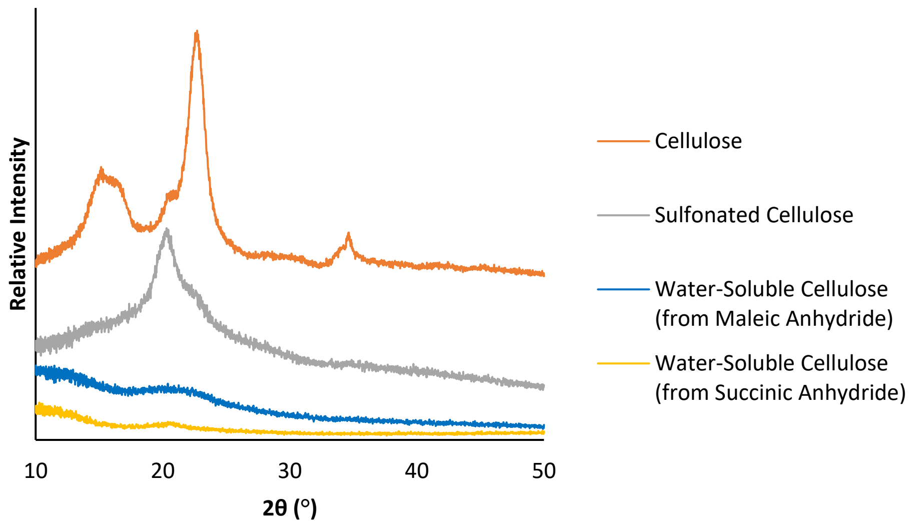

Figure S6: XRD pattern obtained for cellulose, sulfonated cellulose and water-soluble cellulose (Main Paper, Scheme 2).

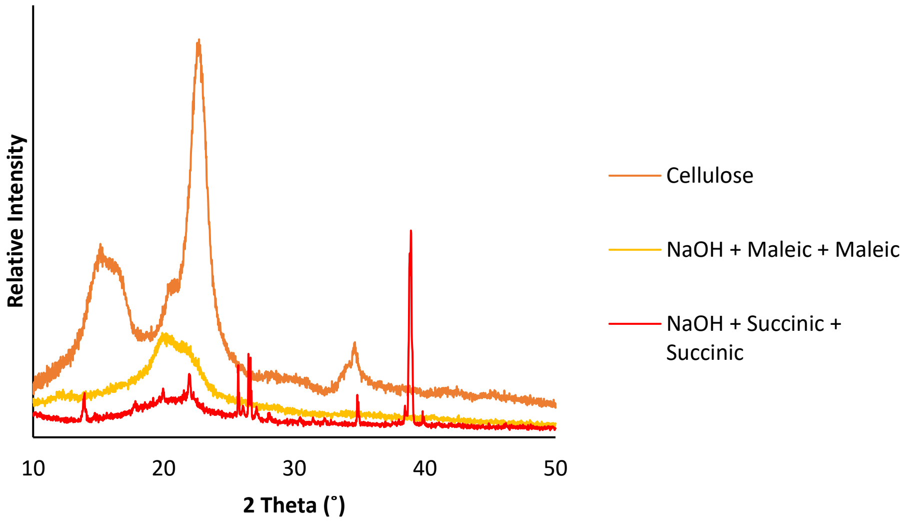

Figure S7: XRD pattern obtained for cellulose, 2-step maleic anhydride modified cellulose (NaOH catalysed), and 2-step succinic anhydride modified cellulose (NaOH catalysed) (See section S2.5) (Main Paper, Scheme 3, top). The succinic acid modified cellulose has a high DS, 1.70) which accounts for the large peaks.

S8 Field Emission Scanning Electron Microscope (FE-SEM)

S8.1 Cellulose

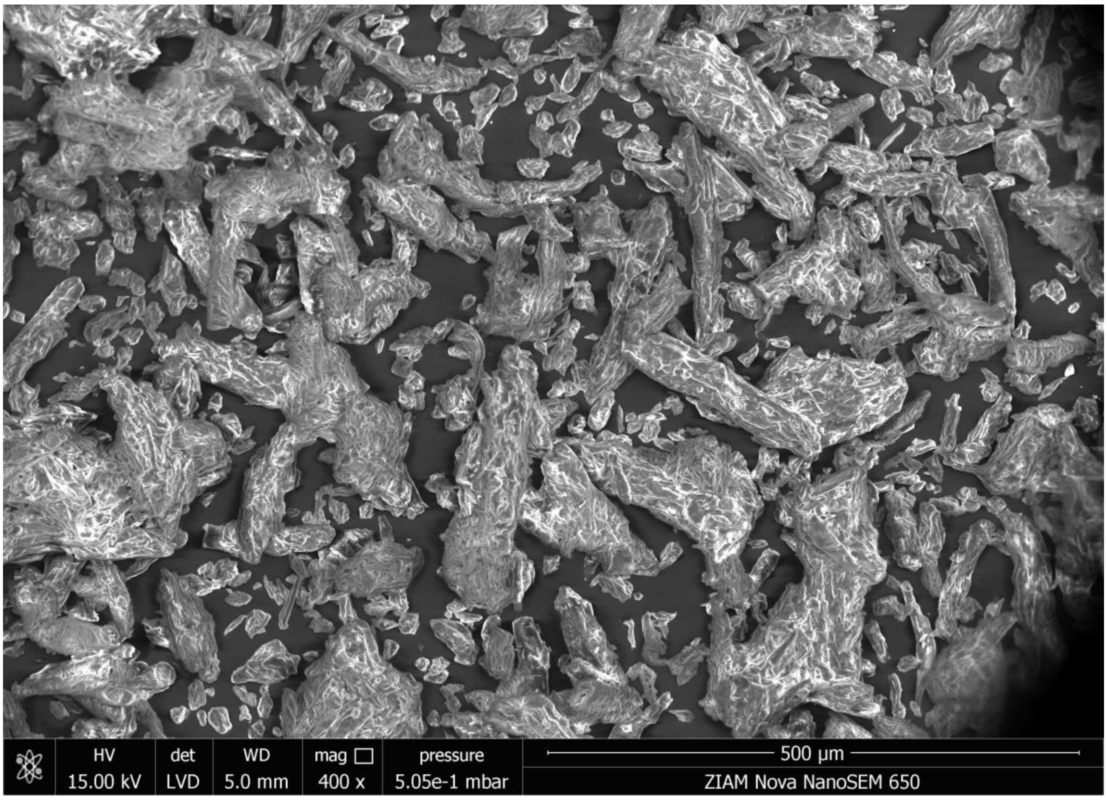

Figure S8: Low vacuum FE-SEM image of cellulose at 400 x magnification.

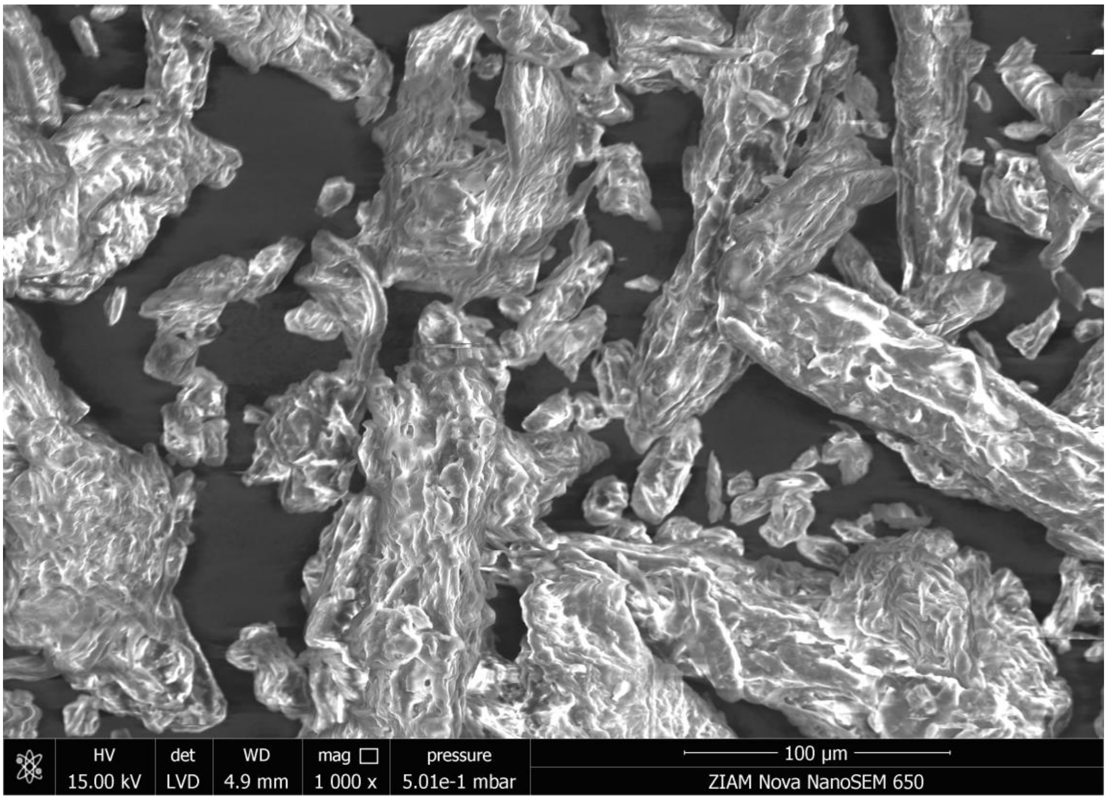

Figure S9: Low vacuum FE-SEM image of cellulose at 1000 x magnification.

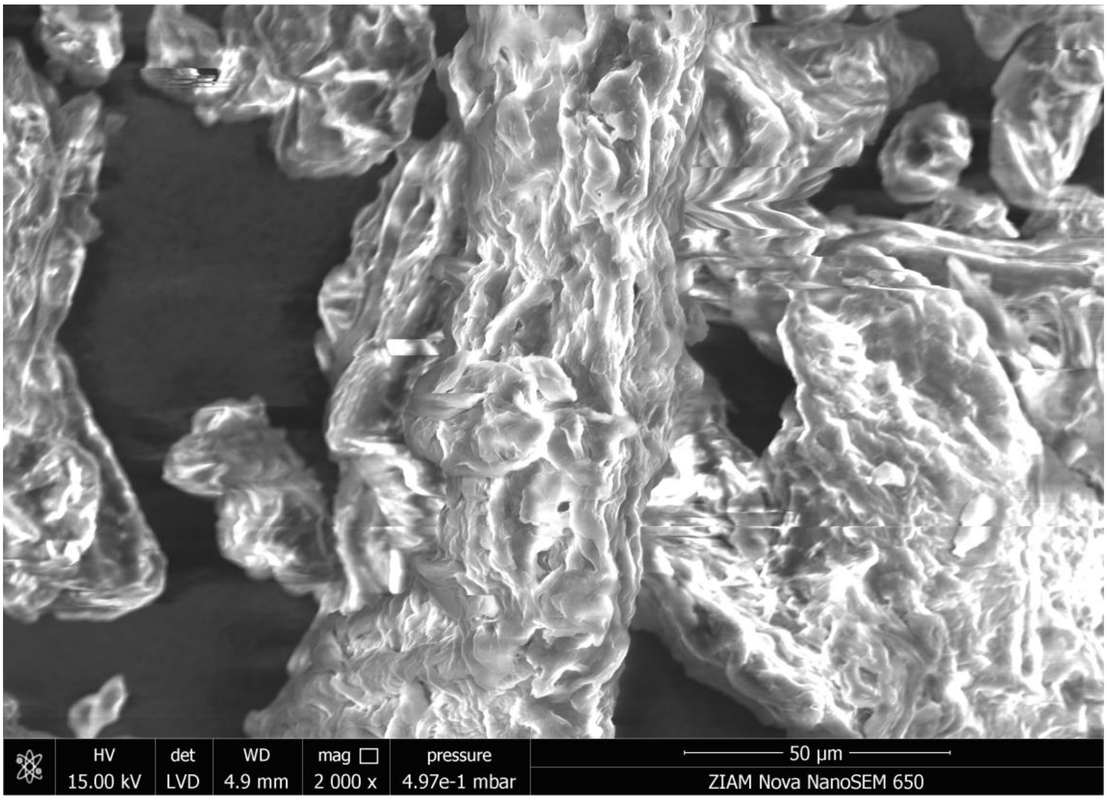

Figure S10: Low vacuum FE-SEM image of cellulose at 2000 x magnification.

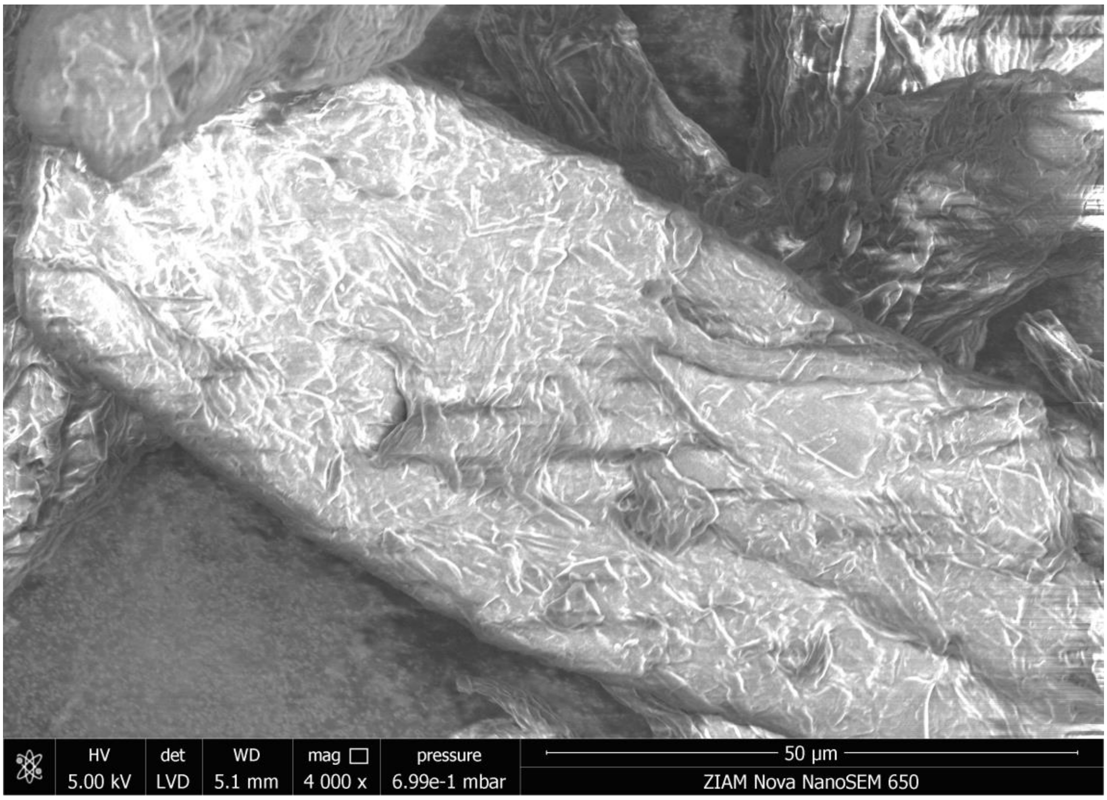

Figure S11: Low vacuum FE-SEM image of cellulose at 4000 x magnification.

S8.2 Sulfonated Cellulose

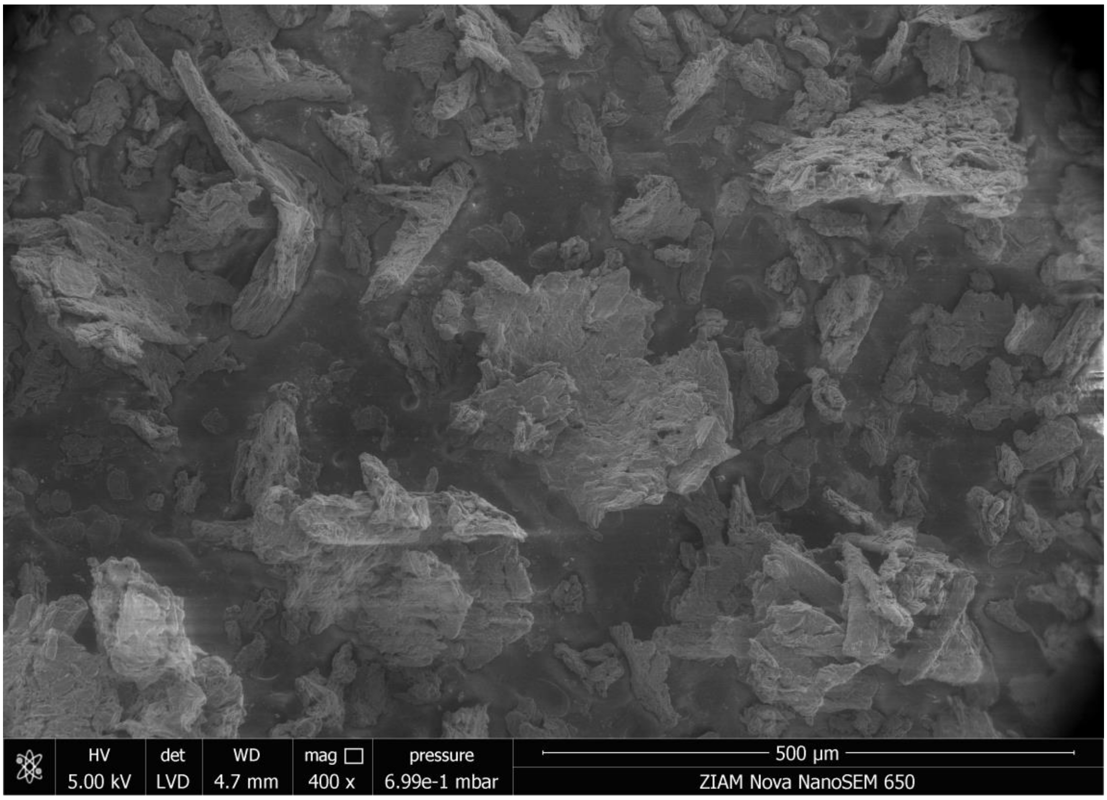

Figure S12: Low vacuum FE-SEM image of sulfonated cellulose at 400 x magnification.

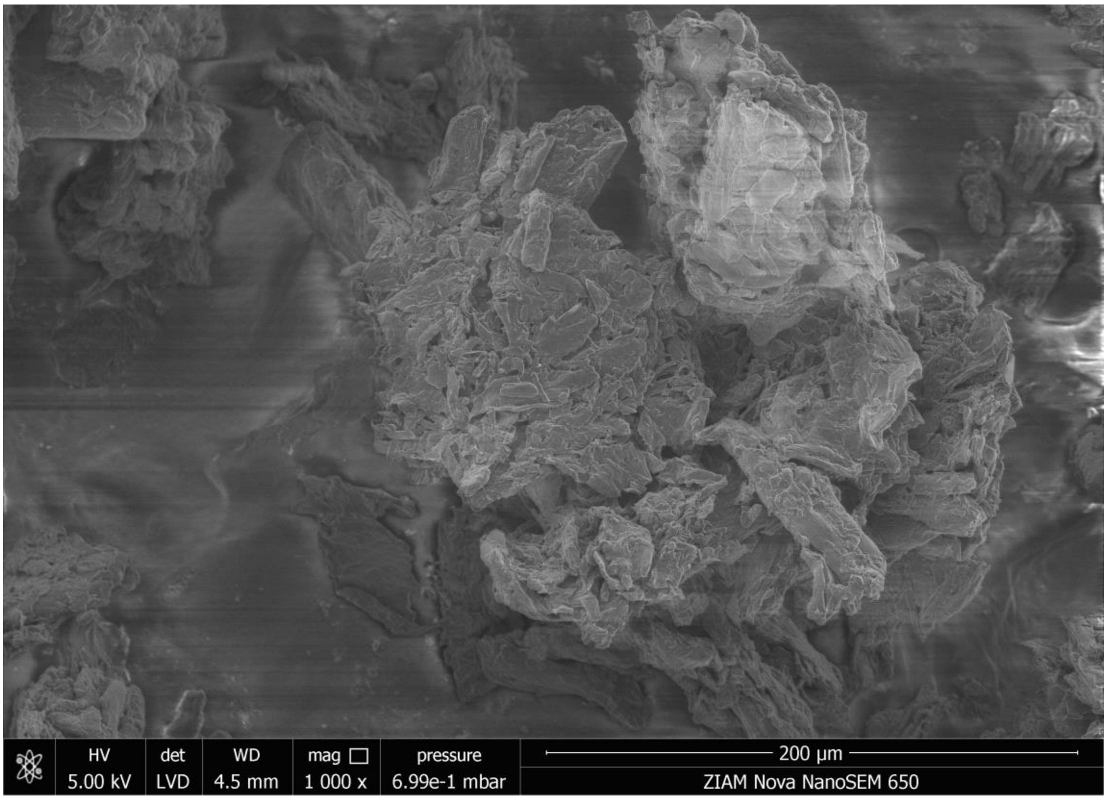

Figure S13: Low vacuum FE-SEM image of sulfonated cellulose at 1000 x magnification.

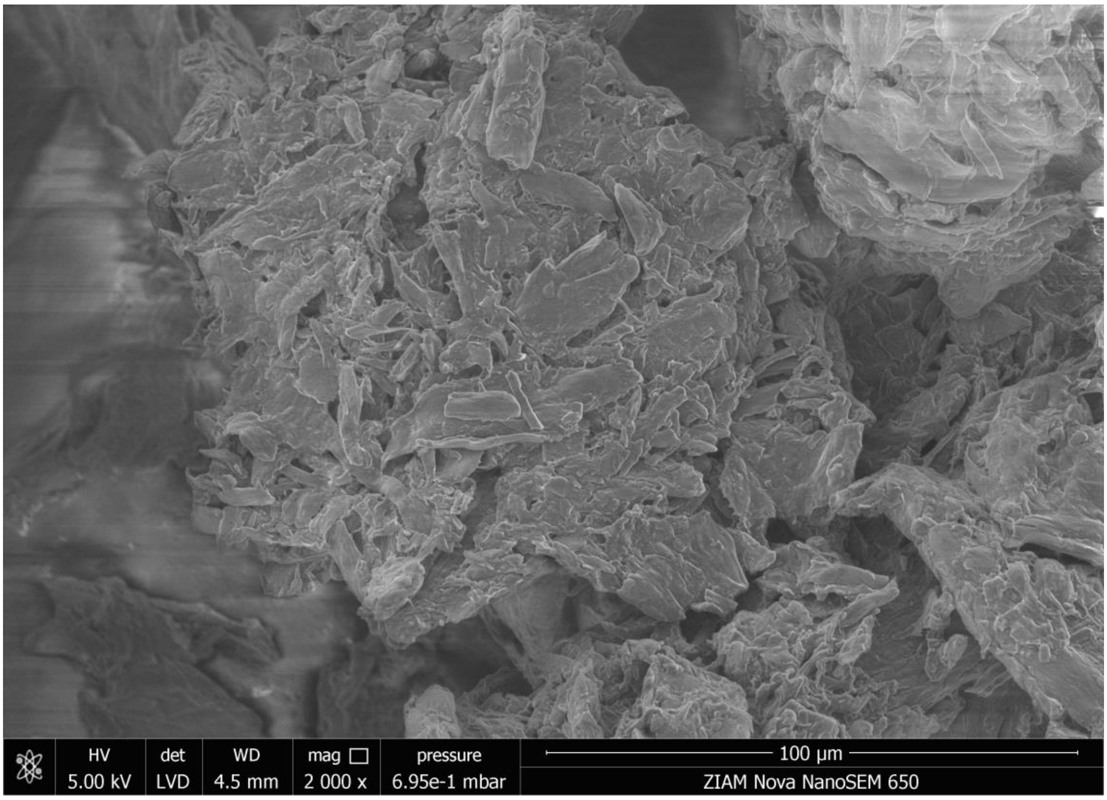

Figure S14: Low vacuum FE-SEM image of sulfonated cellulose at 2000 x magnification.

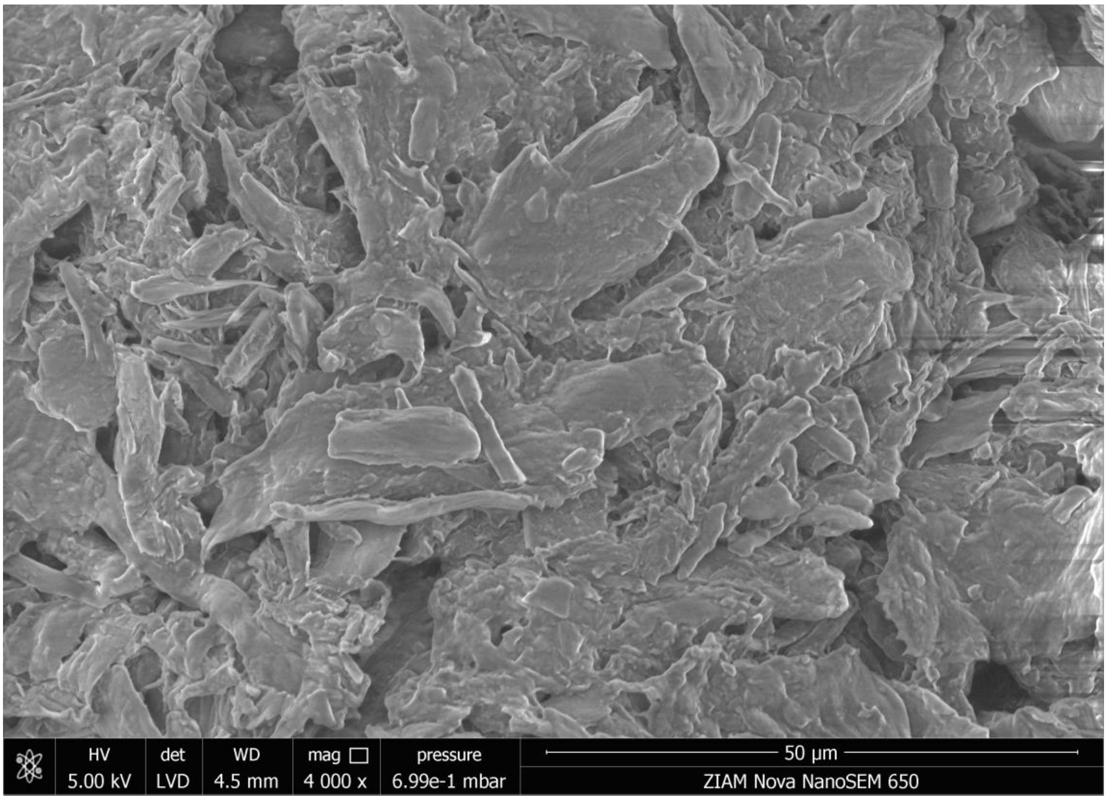

Figure S15: Low vacuum FE-SEM image of sulfonated cellulose at 4000 x magnification.

S8.3 Water-soluble cellulose from 6 h maleic anhydride reaction

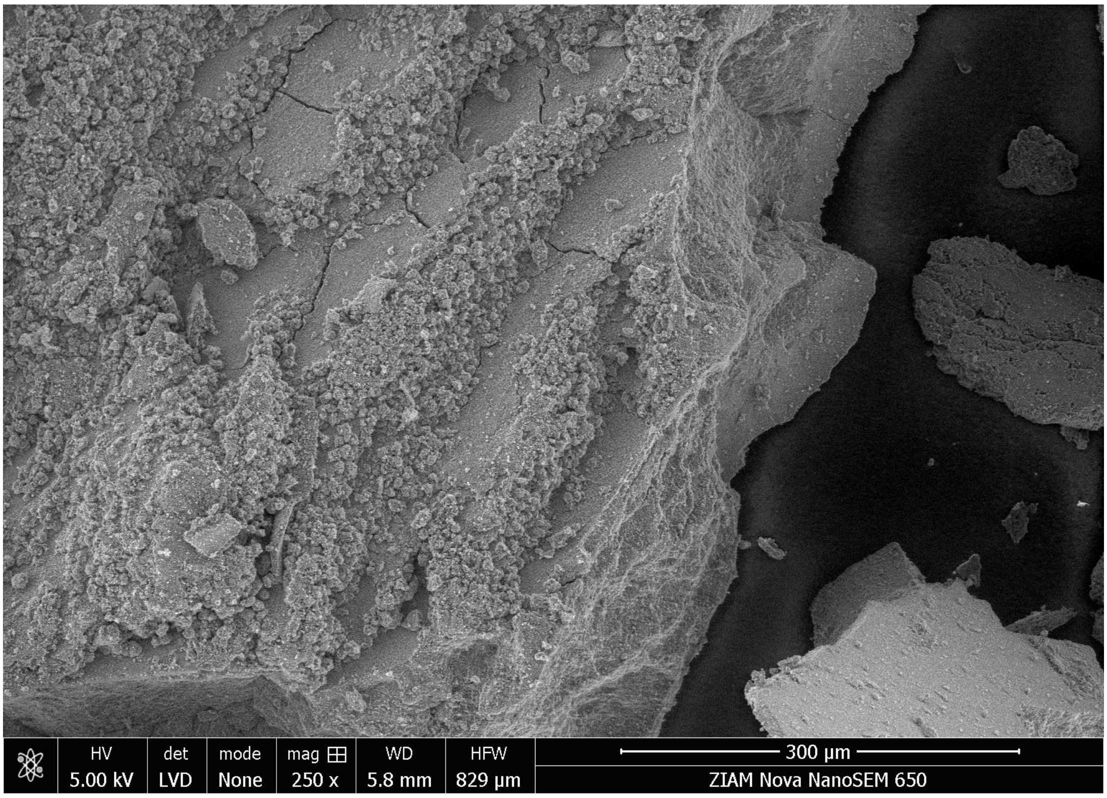

Figure S16: Low vacuum FE-SEM image of water-soluble cellulose from 6 h maleic anhydride reaction at 250 x magnification.

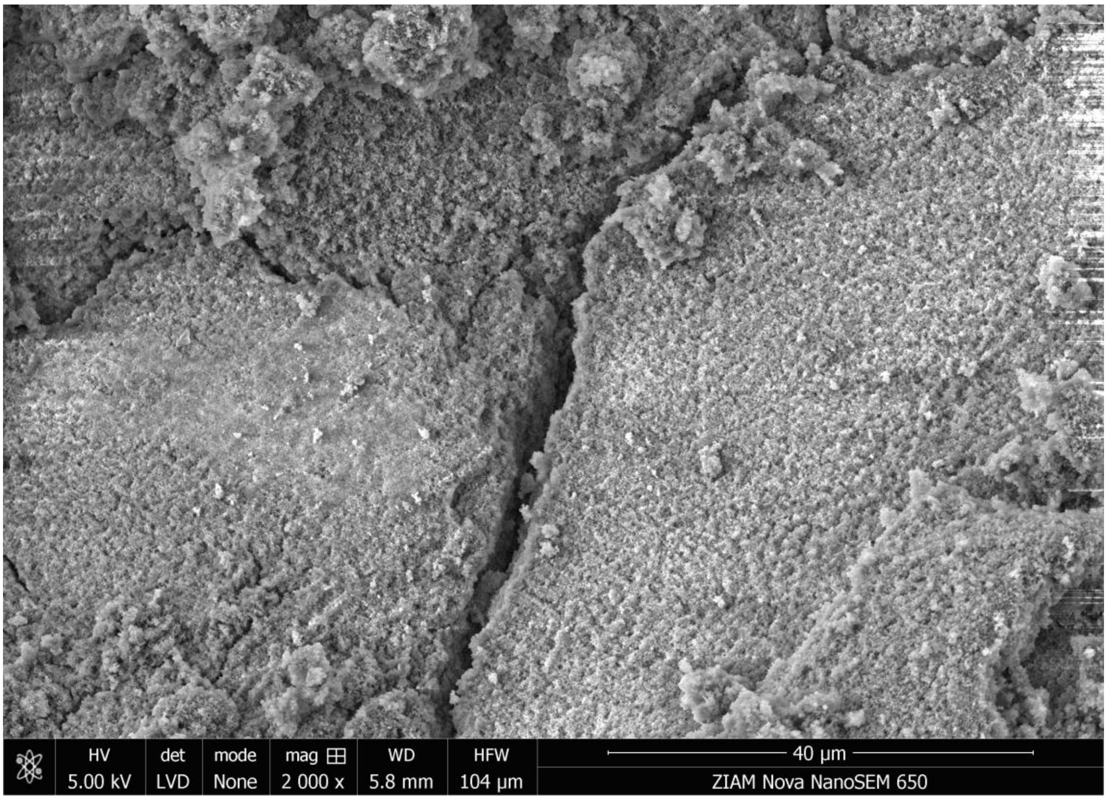

Figure S17: Low vacuum FE-SEM image of water-soluble cellulose from 6 h maleic anhydride reaction at 2000 x magnification.

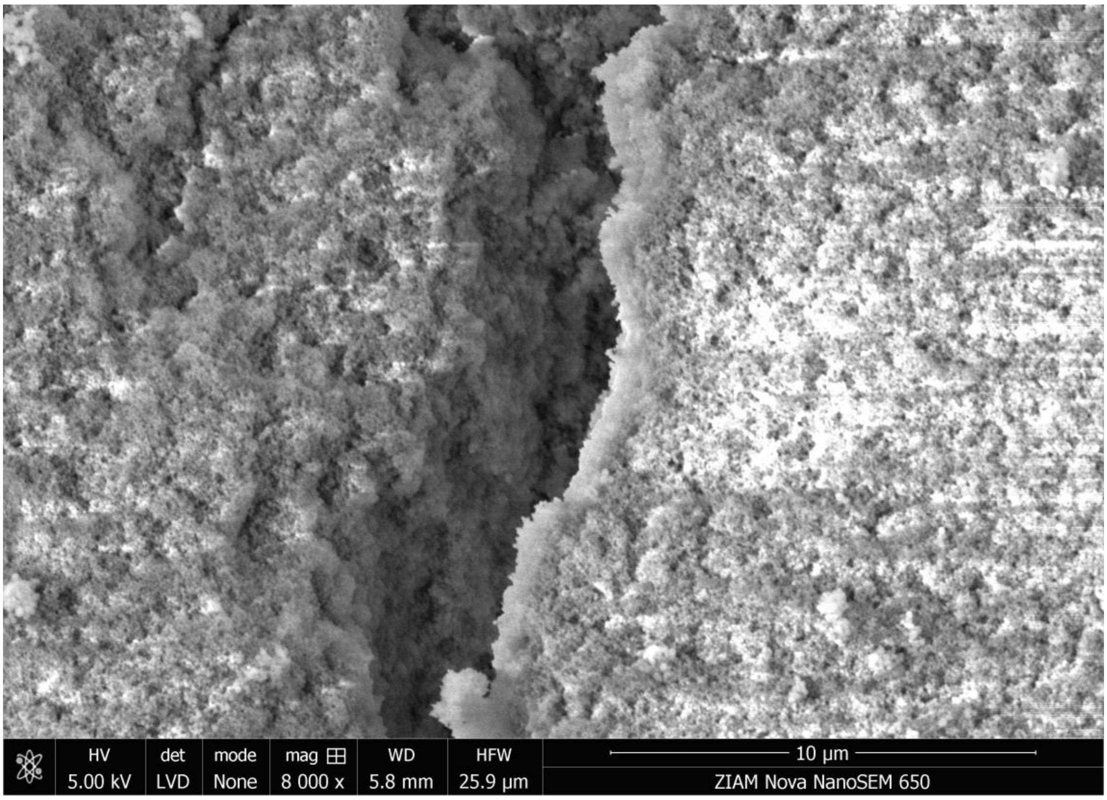

Figure S18: Low vacuum FE-SEM image of water-soluble cellulose from 6 h maleic anhydride reaction at 8000 x magnification.

S8.4 Water-soluble cellulose from 6 h succinic anhydride reaction

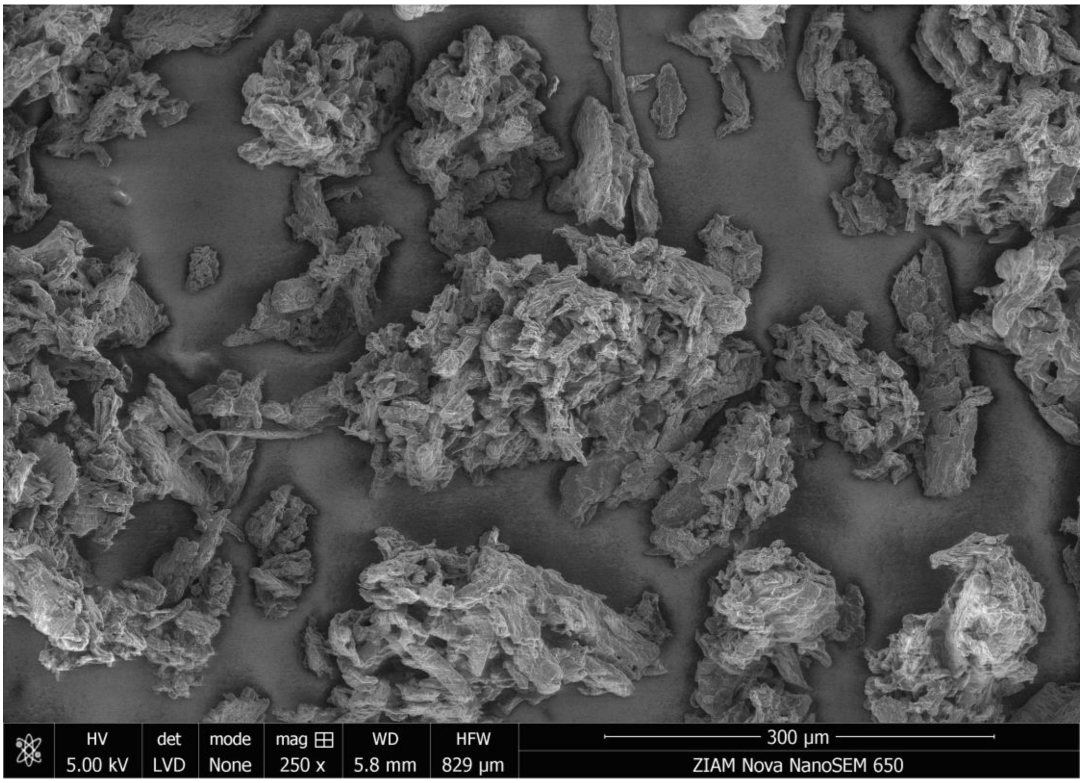

Figure S19: Low vacuum FE-SEM image of water-soluble cellulose from 6 h succinic anhydride reaction at 250 x magnification.

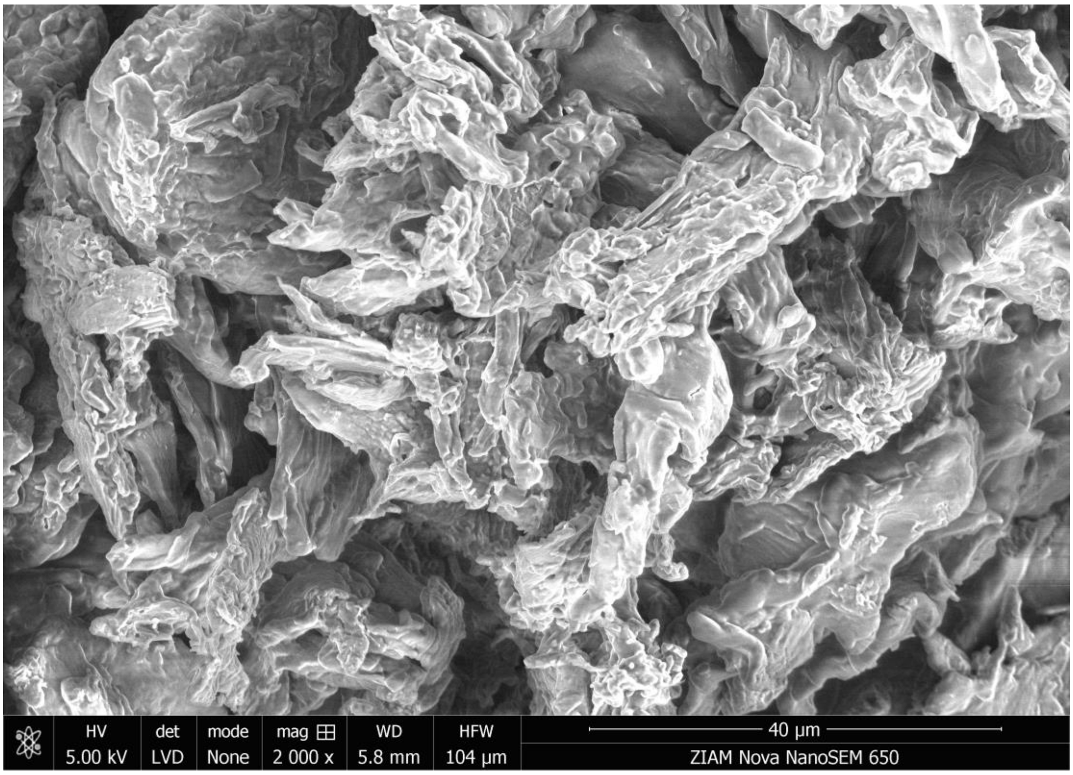

Figure S20: Low vacuum FE-SEM image of water-soluble cellulose from 6 h succinic anhydride reaction at 2000 x magnification.

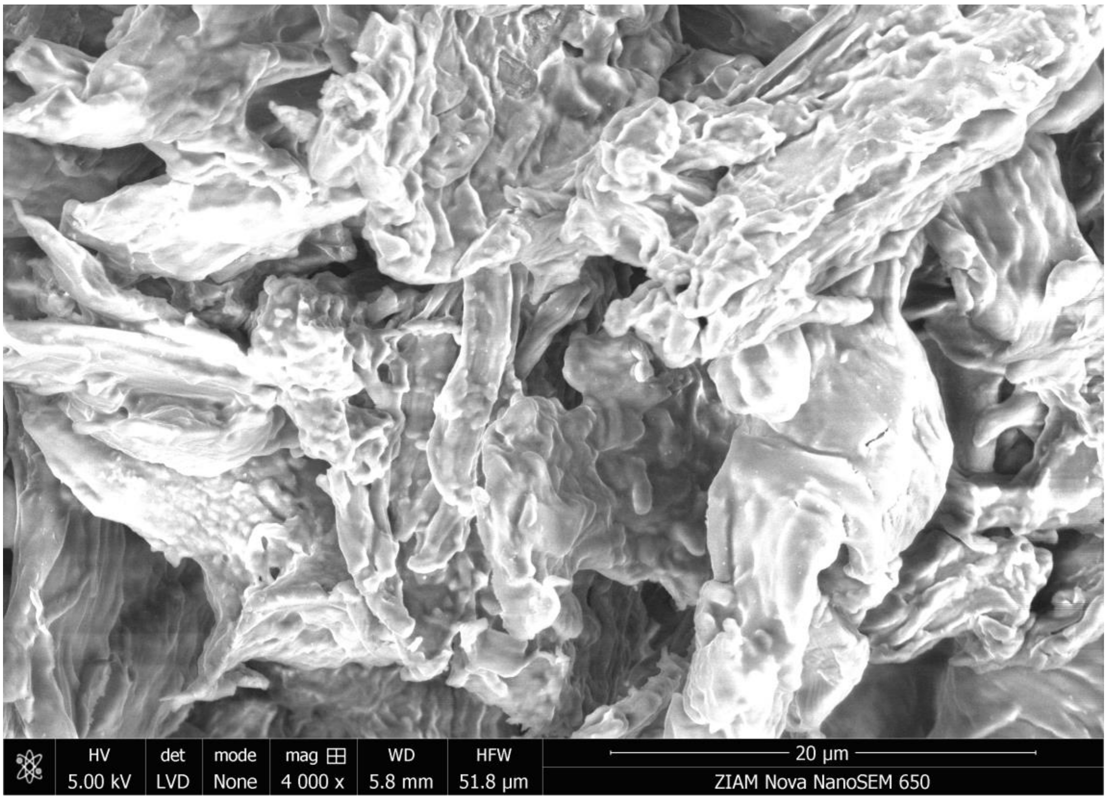

Figure S21: Low vacuum FE-SEM image of water-soluble cellulose from 6 h succinic anhydride reaction at 4000 x magnification.

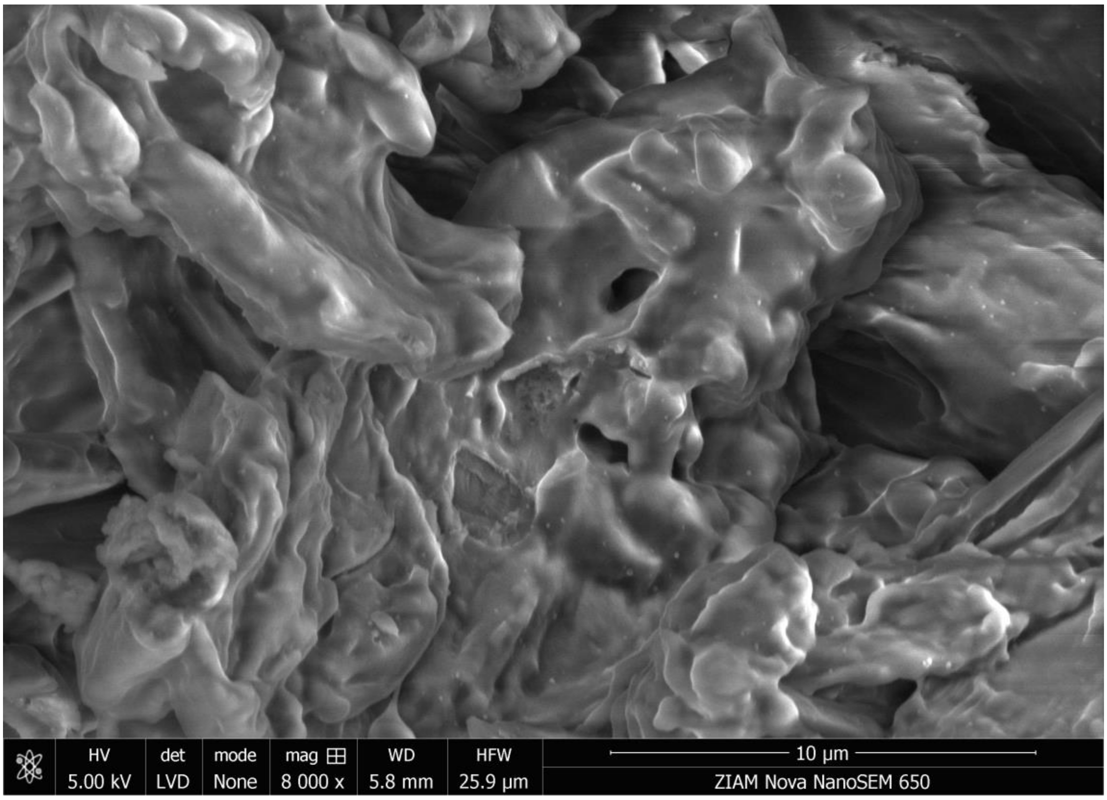

Figure S22: Low vacuum FE-SEM image of water-soluble cellulose from 6 h succinic anhydride reaction at 8000 x magnification.

S9 Thermogravimetric Analysis (TGA)

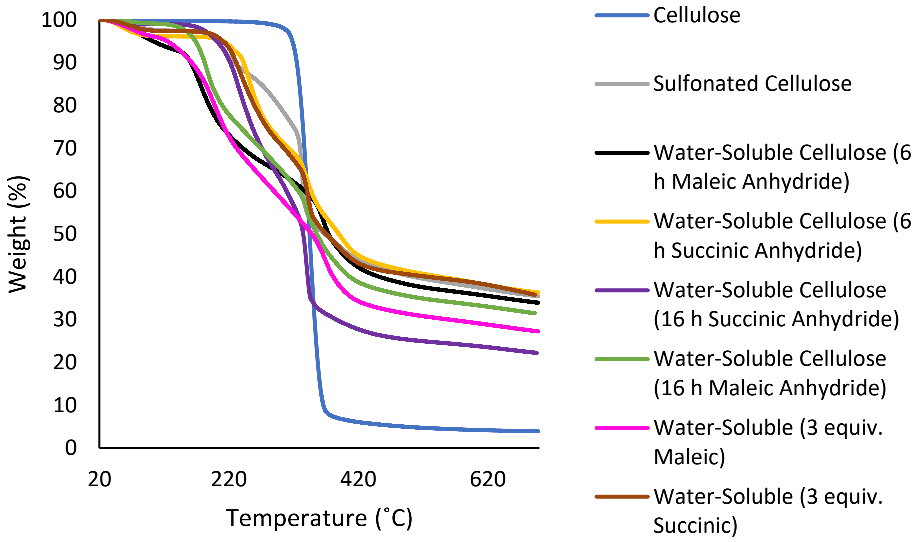

Figure S23: TGA of cellulose, sulfonated cellulose, and water-soluble cellulose analogues.

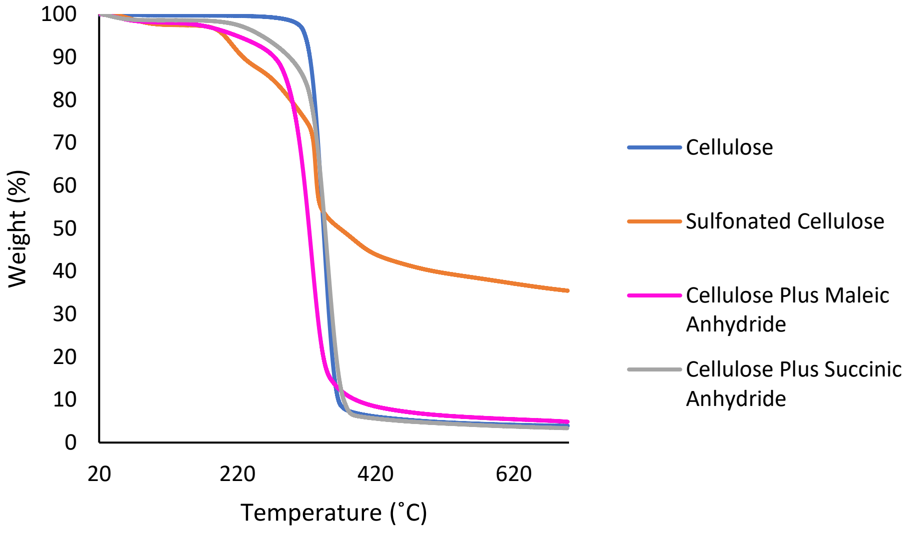

Figure S24: TGA of cellulose and initial cellulose modifications (Main Paper, Scheme 1).

S10 References

[1] M. El-Sakhawy, S. Kamel, A. Salama, H.-A. S. Tohamy, *Cellul. Chem. Technol.*, 2018, **52**, 193-200.
[2] L. Cseri, S. Kumar, P. Palchuber, G. Szekely, *ACS Sustainable Chem.Eng.*, 2023, **11**, 5696-5725.
[3] G. Natus, E. J. Goethals, *J. Macromol. Sci., A*, 1968, **2**, 489-499.
[4] J. C. P. de Melo, E. C. da Silva Filho, S. A. A. Santana, C. Airoldi, *Colloids Surf., A*, 2009, **346**, 138-145.
[5] G. Natus, E. J. Goethals, *J. Macromol. Sci. Part A Pure Appl. Chem.*, 1968, **2**, 489-499.
[6] T. Heinze, O. A. El Seoud, A. Koschella, *Cellulose Derivatives: Synthesis, Structure, and Properties*, Springer International Publishing AG, Switzerland, 2019.
